# A novel approach for power ramps classification in wind generation

**DOI:** 10.1038/s41598-023-48443-4

**Published:** 2023-12-05

**Authors:** M. Saber Eltohamy, M. Said Abdel Moteleb, Hossam E. A. Talaat, S. F. Mekhamer, Walid A. Omran

**Affiliations:** 1 Department of Power Electronics and Energy Conversion, Electronics Research Institute, Cairo, Egypt; 2 Department of Electrical Power and Machines Engineering, Faculty of Engineering, Ain Shams University, Cairo, Egypt; 3 Department of Electrical Engineering, Future University in Egypt, Cairo, Egypt; 4 Department of Mechatronics, Faculty of Engineering and Materials Science, German University in Cairo, Cairo, Egypt

**Keywords:** Energy science and technology, Engineering

## Abstract

Ramp events are characterized by large power changes in a short period and increase with increasing renewable generation. Even with hourly forecasts, their predictions are still unreliable. Thus, grid operators should classify these power ramps to understand their expected occurrence periods and range to balance them. Previous research was based on a binary classification of ramp events, which assumed that ramp events were similar to one another, which is not true. Some other studies used randomization and non-causative classification methods. Hence, a more accurate method is still needed. The paper presents two new methods for ramp event classification. The first method depends on the standard deviation score, and the second method assigns a score to each ramp, which depends on the maximum value of the historical power ramps that occurred within the studied time period. The new classification methods are applied to the output power of Belgium’s aggregated wind farms from 2015 to 2019, and the relative frequency of each ramp category is determined. The results revealed that, even though the capacity of wind installations has doubled, ramping behaviour is nearly the same in all years.

## Introduction

### Motivation

The need to reduce CO2 emissions and other pollutants, as well as events that had a significant impact on fuel supply to the electricity sector and rising energy costs, such as the Coronavirus disease 2019 (COVID-19) and the ongoing war between Russia and Ukraine, have led governments to make decisions to reduce their reliance on fuel, resulting in a rising trend toward renewable energy^1^. As the share of wind and PV generation increases, fluctuations in their power output will have a negative impact on power system operation. The power systems may have not enough flexibility to meet power ramps in both variable renewable generation (VRG) and system demand^2,3^. Flexibility means the ability to cope with variability and uncertainty in generation and demand while maintaining a satisfactory level of reliability at a reasonable cost^4,5^. In Ref.^6^, the important factor in flexibility is the power ramps, and the flexibility requirements were determined by the VRG share and its power ramping behaviour. While wind and PV generations are largely dependent on wind speed and solar irradiance respectively, both speed and irradiance variations cannot fully represent the variability of power^7,8^. In Refs.^9,10^, the authors illustrated that wind speed is less intermittent than wind power, and diverse types of wind turbines react differently to the same wind speed. Moreover, the most recent VRG forecasts are still inaccurate^11^, and forecasting methodologies continue to evolve^12,13^. Due to the wide variety of recorded events and the VRG’s stochastic character, it is difficult to identify and classify power ramps, and getting the range and the level of power ramps is becoming vital information^14,15^. The authors outlined in Refs.^16,17^ that the prediction of severe upward or downward ramp events in wind power that occurred in a short period is very important to ensure the security and reliability of a system and the control actions differ according to the range not the exact value of ramp event. For example, the control action applied to the 250–300 MW/10 min ramp events may be identical. Therefore, the ramp event “class” is considered significant to the power system operators. The majority of previous research on this subject was primarily based on a binary classification of ramp events, which assumes that ramp events are equally identical^18^, which is not true. Additionally, there is no agreement on a precise definition of the ramp event. The other studies were based on an arbitrary classification. In which, a randomized, non-causative classification was used. Accordingly, a more accurate classification method is required.

### Literature review

With the increasing share of VRG, the planning of power systems has been changed to find flexible power resources complementing VRG instead of simply finding power resources to cover consumption^19^, these flexible power resources should have high ramping capabilities in both directions (upward and downward), rapid response, fast start-up and shut-down time to cope with the fluctuations and uncertainty of VRG. In Refs.^20,21^, the output power of a 2 kW PV system can decrease by 50% in just three seconds and also can increase at a similar rate as passing clouds. For scenarios with high PV penetration, these rapid ramps may be more than what the power system utilities can tolerate. In Ref.^22^, the daily capacity factor of wind farms ranges from 5 to 70%. So, retaining more than the nominal operating reserves is highly important to preserve the power balance in the system^11^. Different control actions have been taken by the system operator to deal with upward and downward ramp events. Small ramps have been managed by generation control mechanisms, while large ramp events have been mitigated either by re-dispatching the system or, by shedding the load. The identification of ramp events in historical wind generation data over many years is a statistical challenge, as there are no proven criteria for the concept of ramps^23^. So far, there is no agreement on an accepted definition or categorization for the power ramps^14,24^. In Ref.^25^, the influence of ramp events on system frequency was studied. The study suggested creating uncertainty boundaries, which depend on looking into the uncertainty from the previous data, to show the likelihood that such deviations may occur^26^. A method for classifying the power ramps in the system should be developed; this method can describe the system ramp events during the period under study and enables the power system operator to easily know the probability of occurrence of each level. The previous classification methods, which were based on binary classification or arbitrary classification of ramp events, are illustrated below.

#### Binary ramp event classification

Many authors have used the binary ramp event classification to classify ramp events. Table 1 below lists a literature review of the binary ramp classification, where the basic function for the binary ramp classification (${I}_{t}$) is illustrated as follows:

1 $${I}_{t}=\left\{\begin{array}{l}1\, if {S}_{t}\ge\, {S}_{0}\\ 0\, if {S}_{t}<{S}_{0}\end{array}\right.,$$

where ${S}_{t}$ is a certain criterion function that is assessed at time t, and ${S}_{0}$ is the threshold value. The criterion function is usually based on the variation in power during a specified time interval^27^. This value represents the amount of change that is difficult to manage during a specified period. Mathematically,

2 $${{S}_{t}=\Delta p }_{t}=P\left(t+\Delta t\right)-P\left(t\right),$$

where ${S}_{0}= {\Delta p}_{0}$ ${\Delta p}_{0}>0$—Threshold value, ${S}_{t}>{\Delta p}_{0}$ ${I}_{t}=1$—Upward ramp event, ${S}_{t}<{-\Delta p}_{0}$ ${I}_{t}=-1$—Downward ramp event.

**Table 1:** The literature review of binary ramp classification.

Author	Ramp type	${\mathrm{\Delta p}}_{0}$	Δt	Type
Ren^7^		${1\mathrm{\%p}}_{{\text{R}}}$	5 min	Wind
		${3\mathrm{\%p}}_{{\text{R}}}$	15 min	
		$7\mathrm{\%}{{\text{p}}}_{{\text{R}}}$	30 min	
		$15\mathrm{\%}{{\text{p}}}_{{\text{R}}}$	1 h	
Sherry^29^		${50\mathrm{\%p}}_{{\text{R}}}$	˂4 h	Wind
Dorado-moreno^10^		${50\mathrm{\%p}}_{{\text{R}}}$	6 h	Wind
Bradford^30^		$20\mathrm{\%}{{\text{p}}}_{{\text{R}}}$	1 h	Wind
Bossavy^26^		$50\mathrm{\%}{{\text{p}}}_{{\text{R}}}$	n/a	Wind
Kamath^31^		10–12% P_R_	30 min	Wind
		15–20% P_R_	1 h	
Gallego^32^		${\upsigma }_{{\text{g}}}{{\text{p}}}_{{\text{R}}}$	1 h	Wind
Cutler^33^		200 MW	30 min	Wind
	Downward	150 MW	5 min	
		150 MW	30 min	
		75 MW	30 min	
Zareipour^17^		50%${{\text{p}}}_{{\text{R}}}$	10 min	portfolio
Girard^34^		${30\mathrm{\%p}}_{{\text{R}}}$	n/a	Wind
Ohba^35^		${30\mathrm{\%p}}_{{\text{R}}}$	˂6 h	Wind
Yang^36^		${15\mathrm{\%p}}_{{\text{R}}}$	1 h	Wind
Fernandez^37^		${25\mathrm{\%p}}_{{\text{R}}}$	3 h	Wind
Suzuki^38^		${15\mathrm{\%p}}_{{\text{R}}}$	6 h	Wind
Revheim^39^		${30\mathrm{\%p}}_{{\text{R}}}$	3 h	Wind
Heckenbergerova^40^		${50\mathrm{\%p}}_{{\text{R}}}$	5 h	Wind
Gan^41^	Upward	${40\mathrm{\%p}}_{{\text{R}}}$	n/a	Wind
	Downward	${30\mathrm{\%p}}_{{\text{R}}}$	n/a	
Wellby^42^		$60\mathrm{\%}{{\text{p}}}_{{\text{R}}}$	1 h	Solar
Abuella^28,43^		0.4 per unit	1 h	Solar
Cui^44^	Upward	${20\mathrm{\%p}}_{{\text{R}}}$	4 h	Wind (Solar)
	Downward	$15\mathrm{\%}{{\text{p}}}_{{\text{R}}}$		
	Upward	${5\mathrm{\%p}}_{{\text{R}}}$	4 h	Load (net-load)
	Downward	${7\mathrm{\%p}}_{{\text{R}}}$		

Table 1 illustrates the following:

1. A wide range of different threshold ramp values that used for ramp classification, as the threshold values ranged from 1 to 60% of the rated power (P_R_), and the considered time interval varied between 5 min and 6 h.
2. Ramps classified as ramp events were similar to each other, while in reality ramp events have different characteristics and different operation strategies are used to deal with them.
3. The number of ramp events identified in these studies was highly sensitive to the selected threshold value^28^, which increased the difficulty of comparing results from different analyses^23,27^.

#### The arbitrary classification of ramp events

In some of the previous studies, a randomized and non-causative classification was used to classify ramp events into several levels. The authors of Ref.^14^ arbitrarily categorized upward and downward ramp rates based on their duration into short, medium, and long durations. The results, which were based on a dataset for a limited 2-year period, demonstrated that there is no identifiable diurnal pattern in the short-duration ramps, and there is no recognizable cycle at any scale across the different months of the year. The authors illustrated the need for more research to assess the generality of these findings. In Ref.^23^, based on the standard deviation for each year, the three standard deviation ramp threshold was selected as the appropriate benchmark ramp definition, where the author assumed that the distribution of the data was Gaussian, which is not true. The author stated that more robust statistical techniques could be used to improve the definition. The author in Ref.^45^ tried to classify ramp events that occurred within the time intervals of 15, 30, and 60 min. For each time interval, three threshold values were selected to classify the severity of power ramps into low, moderate, and high. These fixed threshold values were chosen and experimented with various values to demonstrate the sensitivity of the results to the choice of the thresholds. The author stated also that in practice, this threshold value should be determined according to input from system operators and schedulers, and it could change in value from one region to another or change for the same region by changing the generation mix. The authors in Refs.^16,17^, applied data mining for classifying wind power ramps, where the historical data was used in the classification of power ramps according to pre-defined threshold values, and arbitrary ramp threshold values were chosen to classify the ramp events in four categories from C1 to C4 by using support vector machines in Ref.^17^, while in Ref.^16^ the ramp events were classified into three categories from C1 to C3 by using extreme learning machines. In Ref.^46^, the authors analyzed the variability of wind power in multiple regions using historical real data of many years, where the regions analyzed were classified into three categories: low, medium, and high variability according to the maximum one-hour ramps. In low-variability regions, the maximum ramps in 1 h were below 10% of the rated capacity, while in high-variability regions they were close to 30%.

Because of this heterogeneity in the classification of ramp events, a unified framework for solving this complexity is required. The paper presents a novel approach for ramp classification by which the power system operators or planners can get easily information about the expected ramp events in case of large forecasting errors and take the necessary precautions to balance these ramps. The following is the structure of this paper: In “The proposed ramp classification methods” section, the paper demonstrates the characteristics that should exist in a classification method before explaining the two proposed methods based on these characteristics, it also demonstrates how to calculate the relative frequency of each ramp category. “Case study (Belgium’s aggregated wind farms)” section clarifies the data and information of the case study to which the two proposed methods will be applied, where the two proposed methods are applied to real data collected from aggregated wind farms between 2015 and 2019. In “Results and discussion” section, the results are displayed. Finally, in “Conclusion” section, the paper summarizes the major findings. The following points summarize the paper’s contribution:

1. Introducing two new techniques for classifying power ramps in variable renewable generation. The two new methods overcome the drawbacks of previous techniques, avoiding heterogeneity between different energy systems in defining the ramp event, and introducing a unified framework for solving this complexity.
2. The first technique revealed the nearly constant relative frequency of each ramp category; however, this classification is dynamic, as it is a function of the average and standard deviation. As a result, a severe power ramp 1 year may become a medium power ramp the next.
3. The second classification method complements the first one for high standard deviation values because the power ramps’ magnitudes extend over a wider range and their average value does not perfectly express the ramps in the system. As a result, the second method assigned each ramp a degree, indicating its intensity.
4. The new classification techniques’ applicability to different time horizons provides detailed information on the level of the power ramps at each observation time, month, season, or year. This information includes the relative frequency of a specific class or degree of power ramp, which can be used to estimate the probability of occurrence of different categories of power ramps, as well as the times when high and severe ramps are most likely to occur, allowing the system to take the necessary precautions of operating reserves to balance these ramps in the event of a significant forecast error, enhancing system flexibility.

## The proposed ramp classification methods

As a result of the previous literature, a more accurate classification method will be needed to solve the heterogeneity in the previous classification methods. This method should have the following characteristics:

- Capability to distinguish between ramps with different characteristics.
- Considering that the power system utilities have different characteristics. Therefore, each power system utility has a specific ramp threshold value.
- Generality, which means that it should be appropriate for all power systems and not restricted to a specified system.

According to the preceding characteristics, two proposed methods for ramp classification will be presented in the following subsections, the power ramps are classified within the selected time interval that is chosen by the power system operator based on the studied operational stage (regulation or load following), and that this time interval remains constant throughout the calculations.

### Power ramp classification technique based on standard deviation score

The concept of standard deviation (σ) has been used in probability calculation to predict the range of future outcomes by using historical data. Even if the results are not certain, there is a high probability degree that the results will be reasonably acceptable and the certainty degree rises with increasing historical data. In VRG such as wind generation, the average value of power ramps $({\mathrm{\Delta p}}_{{\text{avg}}})$ is close to zero due to the continuous fluctuation of power between ramps up and down all the time^15^, so the historical power ramps within the selected time interval Δt are classified into upward and downward then for each ramp type, the average value and the standard deviation are calculated to determine the ramp level, where each power ramp will get a standard score that represents the number of standard deviations in which the magnitude of the power ramp in the selected time interval Δt is above or below the average value of power ramps.

3 $${\Delta p}_{t}= Z,$$

where (${\mathrm{\Delta p }}_{t}$) is the power ramp at observation time t, and Z is the standard deviation score.

This categorization method converts the existing data into a new unit of measurement, which is the standard deviation unit. The average value of power ramps $({\mathrm{\Delta p}}_{{\text{avg}}})$ will have a score of zero (it is zero steps away from itself) and the standard deviation will have a score of one, as presented in Table 2.

**Table 2:** Standard scores.

Data	Standard scores
$\left|{\Delta p}_{avg}\right|$	0
$\left|\sigma \right|$	1
$\left|{\Delta p}_{avg}+Z\sigma \right|$	Z

After assigning the standard deviation scores to the power ramps, the power ramps in each ramp type are classified into four levels, as shown in Table 3.

**Table 3:** Power ramps classification by standard deviation scores.

Ramp category	Constraint
low	$\left|{\Delta p }_{t}\right|<0$
Moderate	$0<\left|{\Delta p }_{t}\right|\le 1$
High	$1<\left|{\Delta p }_{t}\right|\le 2$
Severe	$\left|{\Delta p }_{t}\right|>2$

### Calculation of standard deviation scores for each ramp type within the selected time interval Δt

The historical power ramps that occurred within the time interval Δt are calculated from historical power readings as follows:

4 $${\Delta p}_{n}=P\left({t}_{n}+\Delta t\right)-P\left({t}_{n}\right), n = \left(1, \dots , N\right),$$

where N is the total number of historical readings, then the power ramps are divided into upward and downward; if ${\Delta p}_{n}$ is positive, it refers to power ramp-up ($\Delta p\uparrow$). If ${\Delta p}_{n}$ is negative, it refers to power ramp-down ($\Delta p\downarrow$), which can be expressed as follows:

Initially*, n*_+_ = 0*; n*_*−*_  = 0*; i* = (1, 2, …).

If,

$${\Delta p}_{n}>0\to \Delta p\uparrow \left(i\right), {n}_{+}={n}_{+}+1,$$

and if,

5 $${\Delta p }_{n}<0\to \Delta p\downarrow \left(i\right), {n}_{-}={n}_{-}+1,$$

where ${n}_{+}$, ${n}_{-}$ are counters for upward and downward power ramps, respectively. The average value of upward power ramps (${\Delta p}_{avg}\uparrow$) is given by Eq. (6) as follows:

6 $${\Delta p}_{avgt}\uparrow =\frac{1}{{n}_{+}}\sum_{i=1}^{{n}_{+}}\Delta p\uparrow \left(i\right).$$

The standard deviation of upward power ramps (${\sigma }_{t}\uparrow$)is calculated by Eq. (7) as follows:

7 $${\sigma }_{t}\uparrow =\sqrt{\frac{1}{{n}_{+}-1}\sum_{i=1}^{{n}_{+}}(\Delta p\uparrow (i)-{\Delta p}_{avgt}{\uparrow )}^{2}.}$$

The standard deviation scores of upward power ramps ($Z\uparrow$) are calculated by Eq. (8) as follows:

8 $$Z\uparrow \left(i\right)=\frac{\left(\Delta p\uparrow \left(i\right)-{\Delta p}_{avgt}\uparrow \right)}{{\sigma }_{t}\uparrow }.$$

For the classification of downward power ramps, the average value of downward power ramps (${\Delta p}_{avgt}\downarrow$) is calculated by Eq. (9) as follows:

9 $${\Delta p}_{avgt}\downarrow =\frac{1}{{n}_{-}}\sum_{i=1}^{{n}_{-}}\Delta p\downarrow \left(i\right).$$

The standard deviation of downward power ramps (${\sigma }_{t}\downarrow$) is calculated by Eq. (10) as follows:

10 $${\sigma }_{t}\downarrow =\sqrt{\frac{1}{{n}_{-}-1}\sum_{i=1}^{{n}_{-}}{ \left(\Delta p\downarrow (i)-{\Delta p}_{avgt}\downarrow \right)}^{2}}.$$

The standard deviation scores for downward ramps ($Z\downarrow$) are calculated by Eq. (11) as follows:

11 $$Z\downarrow \left(i\right)=\frac{\left(\Delta p\downarrow \left(i\right)-{\Delta p}_{avgt}\downarrow \right)}{{\sigma }_{t }\downarrow }.$$

Note that:

1. For solar power generation, the diurnal upward power ramping from sunrise to noon, and the downward power ramping from noon to sunset, are not defined as ramp events because the classification method classifies the average magnitude of fluctuations in each time period, which represents the normal daily solar variations, as a low power ramp, as expected.
2. A low standard deviation value means that the magnitudes of power ramps are close to the average value and the average value better represents the ramps in the system, whereas the high standard deviation value demonstrates that the power ramps’ magnitudes extend over a wider range and their average value does not express perfectly the ramps in the system. Consequently, another classification method should be used for high values of standard deviation, as will be illustrated in the next section.

### Power ramp classification technique based on maximum value

In this classification method, a degree from zero to one is given to each ramp within the studied time interval, which indicates the intensity of that ramp with reference to the maximum value of historical power ramps within the same time interval. Therefore, the power ramps are classified according to the ramp intensity factor (RIF) which can be defined as the ratio of the power ramp within the studied time interval Δt to the maximum value of historical power ramps of the same type within the same time interval^47^, which can be expressed by Eq. (12):

12 $$RIF=\frac{{\Delta p }_{t}}{{\Delta p}_{max}}, 0\le RIF\le 1.$$

RIF characteristics are as follows:

- For each ramp type, the values of RIF lie in the interval [0, 1].
- The power system operator can use this factor to measure the intensity of the forecasted power ramp.
- The values of RIF that are close to zero represent very small ramps that can be neglected, while the values close to the extreme point {1} refer to high-power ramps.
- RIF is a time-dependent factor that depends on the studied time interval Δt.
- For solar variations, the intensity degree of the normal daily variations will be low in comparison to the maximum value.

For upward power ramps, RIF can be expressed by Eq. (13):

13 $${RIF}_{\uparrow }=\frac{\Delta p\uparrow (i)}{{\Delta p}_{max+}}, 0\le {RIF}_{\uparrow }\le 1.$$

The maximum historical value of upward power ramps (${\Delta p}_{max+}$) can be calculated from Eq. (5) as follows:

14 $${\Delta p}_{max+}=max \Delta p\uparrow \left(i\right).$$

For downward power ramps, RIF can be expressed by Eq. (15) as follows:

15 $${RIF}_{\downarrow }=\frac{\Delta p\downarrow (i)}{{\Delta p}_{max-}}, 0\le {RIF}_{\downarrow }\le 1.$$

The maximum historical value of downward power ramps (${\Delta p}_{max-}$) can be calculated from Eq. (5) as follows:

16 $${\Delta p}_{max-}=min \Delta p\downarrow \left(i\right).$$

In the two proposed classification techniques, the power system operator can get information about the probability of occurrence of a power ramp with a certain class at an observation time t by Eq. (17) as follows:

17 $$\mathrm{P }\left({N}_{j}\right)=\frac{{N}_{j}}{N},$$

where P(${N}_{j}$) is the probability of occurrence of a power ramp with a certain class (j), ${N}_{j}$ is the number of occurrences of that ramp class at an observation time t and N is the total number of historical readings that are taken into consideration.

## Case study (Belgium’s aggregated wind farms)

Belgium is considered one of Europe’s most interconnected countries, which has the capability for importing and exporting more than 40% of its peak demand. The government intends to close all the nuclear power plants that represent 50% of Belgium’s electricity production, by 2026. For that reason, the installation of wind capacity is growing continuously and nearly doubled in the period from 2015 to 2019. At the end of 2015, the maximum installed wind capacity was 1960.91 MW; while the average installed wind capacity throughout the year was 1849.6 MW. Whereas in 2019, the maximum installed wind capacity was 3796.3 MW and the average installed wind capacity throughout the year was 3506.3 MW. The output power from aggregated wind farms (onshore and offshore) is recorded every 15 min^48^. For that time interval ($\mathrm{\Delta t}$=15 min), the variations in wind power of Belgium’s aggregated wind farms over a period of 5 years from 2015 to 2019 will be determined, and the power ramps will be classified, where each classification method will be applied to the following time horizons:

(i) *Historical data of power ramps at each observation time t* In which the historical data of power ramps at each observation time t within the selected time interval Δt are classified to give the power system planner or operator the information about the levels of power ramps at each observation time. So, in Eq. (4), both t and Δt remain constant, whereas n is altered until all of the examined historical readings at observation time t are completed then moving to the following observation time (t + Δt).
(ii) *Historical data of daily power ramps* In which the historical data of daily power ramps within the selected time interval Δt are classified to get information about the levels of power ramps in certain weeks, months, seasons, or years. In this work, the monthly, seasonal, and yearly classification results are presented.

## Results and discussion

### Classification of power ramps at each observation time t

**Figure 1:**
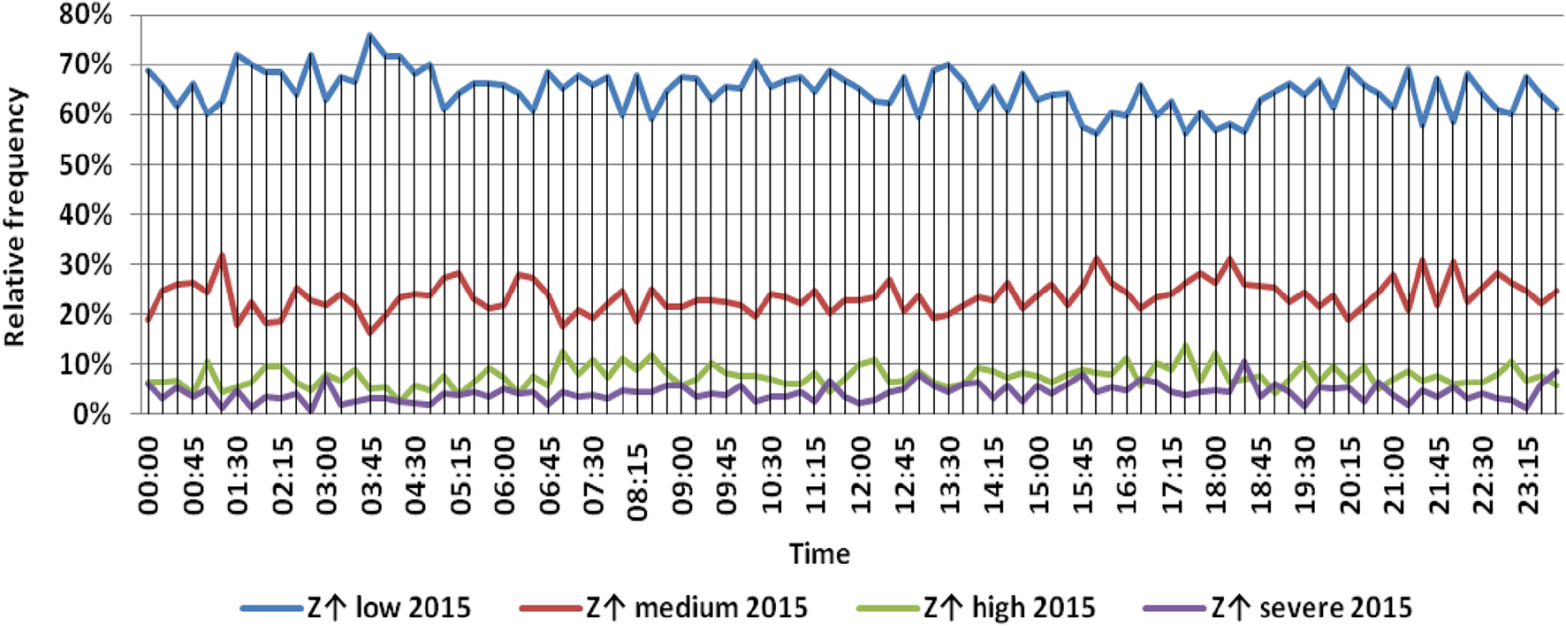
Classification of upward power ramps in 2015 at each observation time according to standard deviation score.

**Figure 2:**
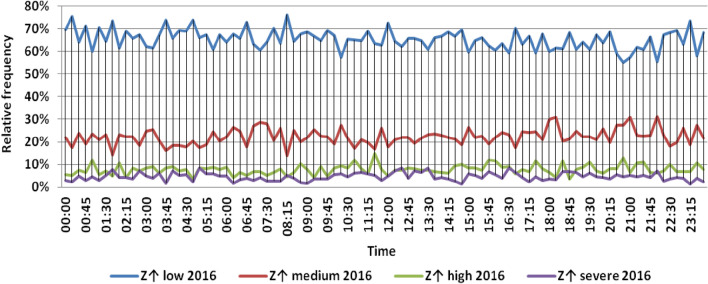
Classification of upward power ramps in 2016 at each observation time according to standard deviation score.

**Figure 3:**
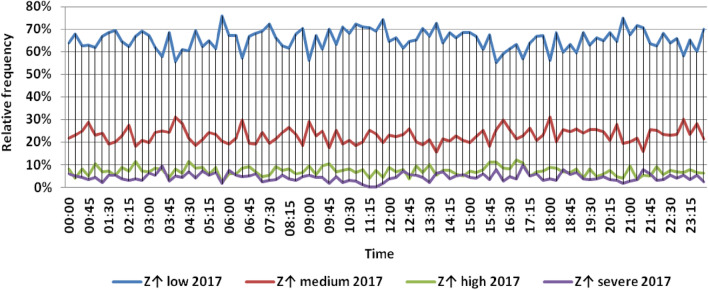
Classification of upward power ramps in 2017 at each observation time according to standard deviation score.

**Figure 4:**
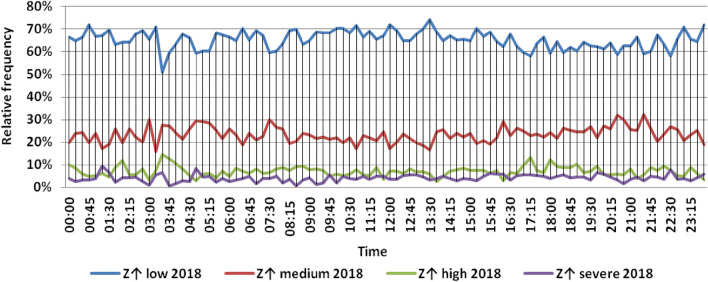
Classification of upward power ramps in 2018 at each observation time according to standard deviation score.

**Figure 5:**
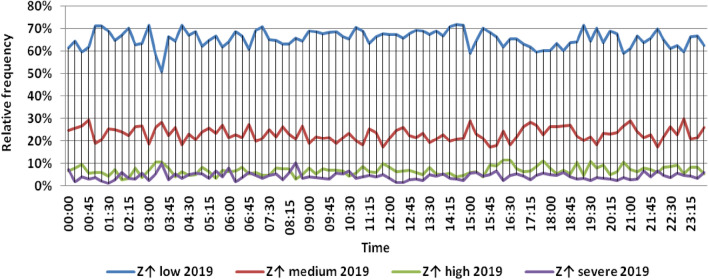
Classification of upward power ramps in 2019 at each observation time according to standard deviation score.

**Figure 6:**
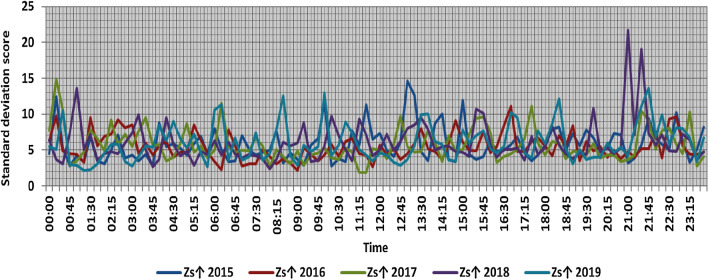
Comparison of the standard deviation scores at each observation time t for maximum upward power ramps.

**Figure 7:**
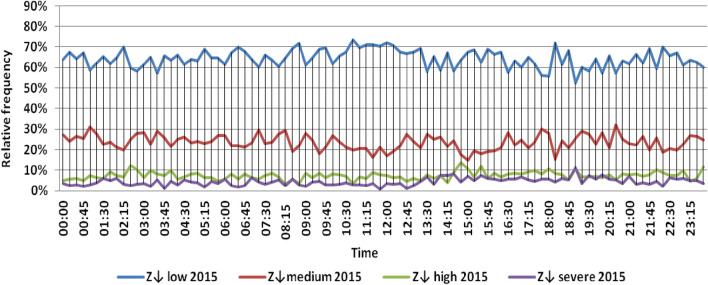
Classification of downward power ramps in 2015 at each observation time according to standard deviation score.

**Figure 8:**
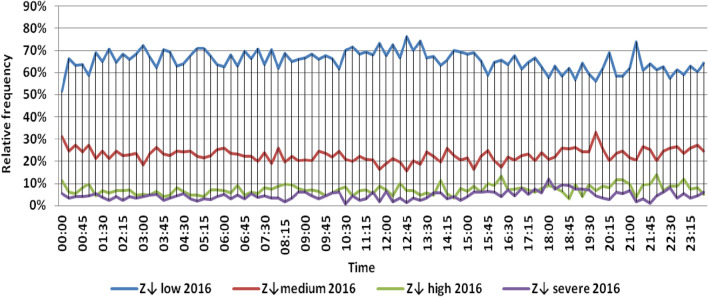
Classification of downward power ramps in 2016 at each observation time according to standard deviation score.

**Figure 9:**
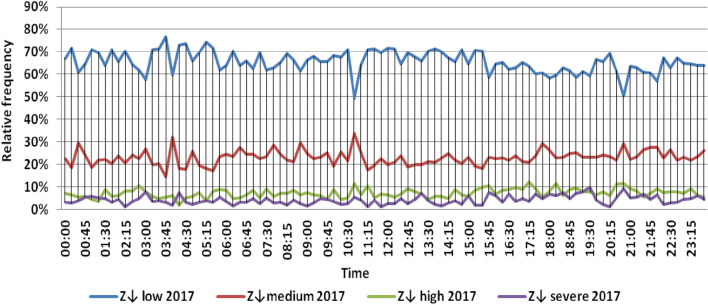
Classification of downward power ramps in 2017 at each observation time according to standard deviation score.

**Figure 10:**
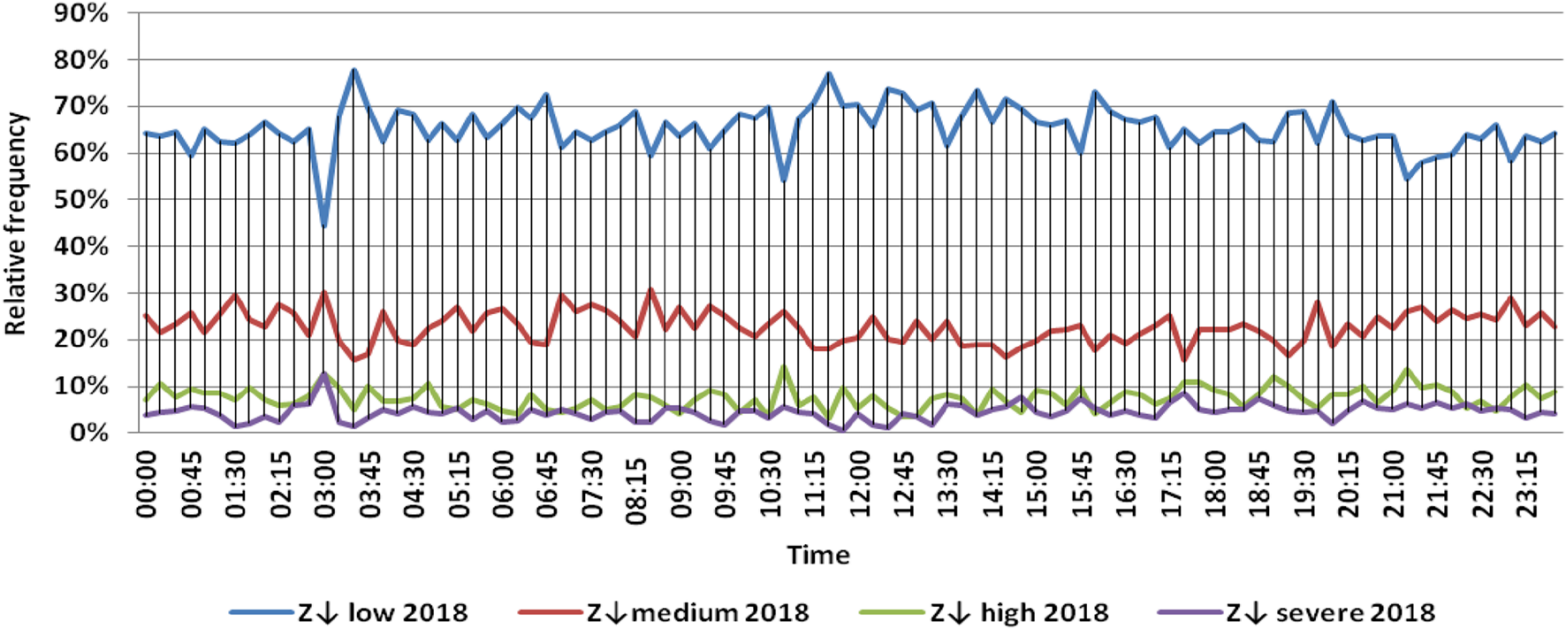
Classification of downward power ramps in 2018 at each observation time according to standard deviation score.

**Figure 11:**
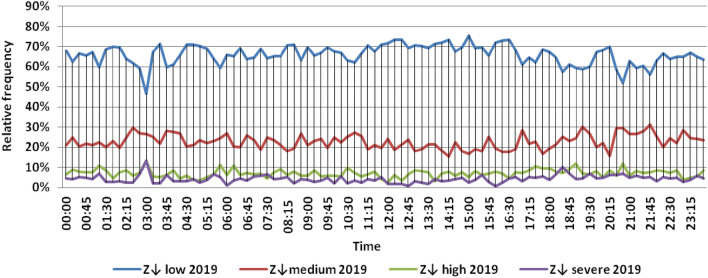
Classification of downward power ramps in 2019 at each observation time according to standard deviation score.

**Figure 12:**
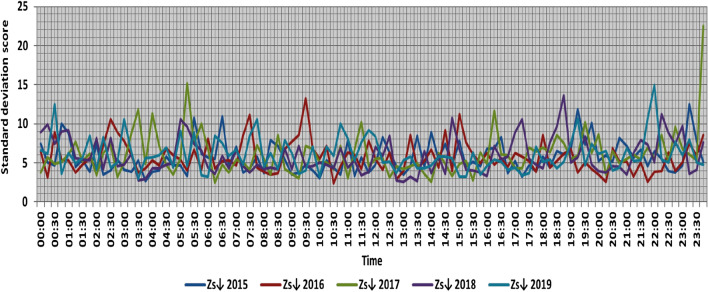
Comparison of the standard deviation scores at each observation time t for maximum downward power ramps.

The classification results illustrated the levels of power ramps in this short time interval at each observation time. The first proposed technique illustrated that approximately 90% of ramps were low and medium power ramps, while severe power ramps did not exceed 5% of ramps. The results in the 5 years are approximately equal. Comparing the relative frequency of each category over the 5 years illustrates that the range and average percentages of each category of power ramps over the 5 years were nearly equal, while the capacity of wind installations was doubled. It also illustrated the following:

(i) For upward power ramps shown in Figs. 1, 2, 3, 4 and 5, low power ramps ranged from 50.96 to 76.16%, with their average percentages ranging from 64.76 to 65.55%. Medium power ramps ranged from 13.9 to 32.3%, with their average percentages ranging from 22.29 to 23.54%. High power ramps ranged from 2.2 to 14.72%, with their average percentages ranging from 6.91 to 7.75%. Severe power ramps ranged from 0 to 10.45%, with their average percentages ranging from 4.14 to 4.57%. Severe power ramps were more common between 2:45–3:30, 12:30–13:30 and 18:30. In Fig. 6, the comparison of the standard deviation scores of maximum upward power ramps at each observation time in the 5 years is shown. The magnitude of the local maximum values of upward power ramps varies significantly between observational times, as shown by the range of their standard deviation scores in Table 4. The severe upward power ramps occurred mostly in the early morning and late evening.
(ii) For downward power ramps shown in Figs. 7, 8, 9, 10 and 11, low power ramps ranged from 44.36 to 77.86%, with their average percentages ranging from 64.47 to 66.04%. Medium power ramps ranged from 14.67 to 34.05%, with their average percentages ranging from 22.75 to 23.64%. High power ramps ranged from 1.69 to 14.29%, with their average percentages ranging from 7.04 to 7.45%. Severe power ramps ranged from 0.56 to 13.44%, with their average percentages ranging from 4.13 to 4.5%. Severe power ramps were more common at 3:00 and between 17:30 and 20:45. In Fig. 12, the comparison of the standard deviation scores at each observation time t for maximum downward power ramps in the 5 years is shown.

**Table 4:** A 5-year comparison of the standard deviation scores of maximum upward ramps at each observation time t.

Year	Range and average of standard deviation scores of maximum upward power ramps overall observation times		
	Range		Average
	From	To	
2015	2.7	14.6	5.8
2016	2.2	11.1	5.4
2017	1.8	14.9	5.7
2018	2.3	21.7	6
2019	2.1	13.7	6

Table 5 summarizes the range and the average relative frequency of each category of power ramps according to the standard deviation scores.

**Table 5:** The range and the average relative frequency as a percentage of each ramp category according to the standard deviation score classification.

Ramp class	2015			2016			2017			2018			2019		
	Avg.	Range		Avg.	Range		Avg.	Range		Avg.	Range		Avg.	Range	
		From	To		From	To		From	To		From	To		From	To
Upward power ramps															
Low	64.76	56.31	75.78	65.39	55.06	76.16	65.47	55.35	75.82	65.37	51.11	74.27	65.55	50.96	71.88
Medium	23.54	16.15	31.65	22.29	13.91	31.29	22.91	15.70	31.21	23.38	15.72	32.32	23.16	17.33	30.05
High	7.46	2.52	13.62	7.75	3.47	14.72	7.23	2.20	11.92	7.11	2.51	14.67	6.91	2.99	11.65
Severe	4.24	0.65	10.45	4.57	1.21	8.53	4.38	0.00	9.74	4.14	0.57	9.38	4.38	1.13	10.13
Downward power ramps															
Low	64.47	52.38	73.65	65.47	51.74	76.32	65.63	49.19	76.63	65.37	44.36	77.86	66.04	46.64	75.48
Medium	23.64%	14.89	32.14	22.87	15.79	33.13	23.01	14.67	34.05	22.76	15.58	30.66	22.75	15.38	31.21
High	7.45	3.33	14.01	7.17	3.05	13.90	7.21	1.69	12.15	7.43	3.30	14.29	7.04	2.53	13.44
Severe	4.44	0.60	11.31	4.50	0.60	12.05	4.13	1.03	9.94	4.44	0.56	12.73	4.18	0.57	13.44

**Figure 13:**
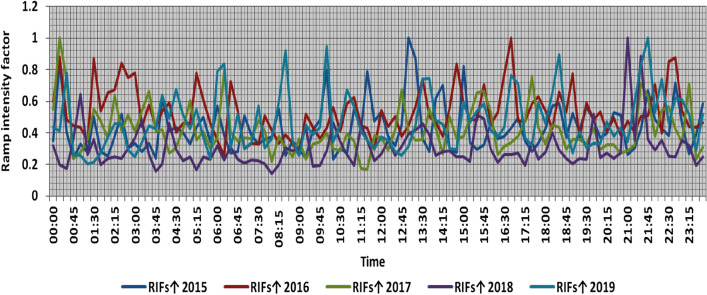
Comparison of RIFs at each observation time t for maximum upward power ramps in the 5 years.

**Figure 14:**
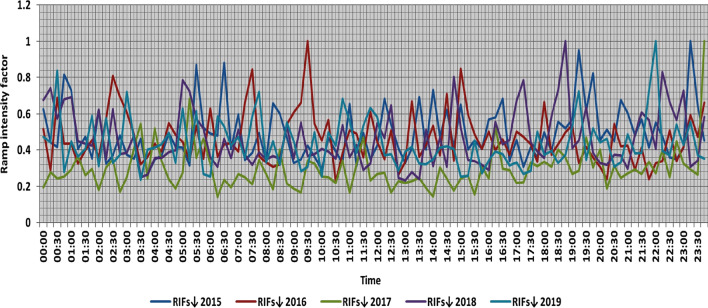
Comparison of RIFs at each observation time t for maximum downward power ramps in the 5 years.

In the second proposed technique, the power ramps with RIF from 0 to 1 are classified into ten categories each 0.1 apart, Tables 6 and 7 present the range and the average relative frequency of each category of RIF for upward and downward power ramps respectively overall observation times in the 5 years, which illustrates that the majority of power ramps have a RIF of less than 0.2. The summation of the average relative frequencies of power ramps with a RIF of more than 0.5 overall observation times is less than 0.4% in the 5 years. Over the 5 years, the summation of the maximum relative frequencies of ramps that have a RIF of more than 0.5 overall observation times is less than 5%. The comparison of ramp intensity factors of maximum upward and downward power ramps that occurred within a time interval of 15 min at each observation time t in the 5 years is shown in Figs. 13 and 14.

**Table 6:** The range and the average relative frequency as a percentage of each category of RIF for upward power ramps overall observation times in the 5 years.

RIF↑ category	2015			2016			2017			2018			2019		
	Avg.	Range		Avg.	Range		Avg.	Range		Avg.	Range		Avg.	Range	
		From	To		From	To		From	To		From	To		From	To
0–0.1	83.34	75.12	90.57	75.06	67.01	84.24	84.33	73.85	93.41	92.70	86.67	96.73	83.40	70.19	90.96
0.1–0.2	13.19	7.45	21.60	17.65	8.28	25.00	12.15	4.95	18.97	6.21	2.22	13.15	12.52	7.78	19.71
0.2–0.3	2.52	0	6.47	4.93	0.61	9.19	2.49	0	5.23	0.80	0	2.53	2.89	0.50	7.21
0.3–0.4	0.58	0	1.99	1.44	0	3.76	0.69	0	2.56	0.20	0	1.99	0.69	0	2.79
0.4–0.5	0.21	0	1.69	0.54	0	2.13	0.19	0	1.60	0.05	0	0.72	0.27	0	1.68
0.5–0.6	0.09	0	1.08	0.22	0	1.49	0.07	0	0.93	0.01	0	0.51	0.11	0	1.29
0.6–0.7	0.02	0	0.60	0.06	0	1.24	0.04	0	0.63	0.01	0	0.66	0.05	0	1.14
0.7–0.8	0.02	0	0.62	0.05	0	0.64	0.03	0	0.58	0	0	0	0.04	0	0.68
0.8–0.9	0.02	0	0.59	0.03	0	0.60	0	0	0	0.01	0	0.51	0.02	0	0.51
0.9–1	0.01	0	0.51	0.01	0	0.50	0.01	0	0.56	0.01	0	0.53	0.02	0	0.63

**Table 7:** The range and the average relative frequency as a percentage of each category of RIF for downward power ramps overall observation times in the 5 years.

RIF↓ category	2015			2016			2017			2018			2019		
	Avg.	Range		Avg.	Range		Avg.	Range		Avg.	Range		Avg.	Range	
		From	To		From	To		From	To		From	To		From	To
0–0.1	78.43	67.86	88.62	81.46	71.08	88.82	93.10	86.25	97.33	81.49	63.27	90.29	85.07	69.57	92.44
0.1–0.2	16.20	7.78	23.68	13.91	8.16	20.98	5.92	1.14	13.13	14.18	6.96	24.00	11.73	5.79	20.16
0.2–0.3	3.62	0	8.93	3.09	0.51	6.92	0.74	0	3.57	3.05	0	9.45	2.22	0	5.76
0.3–0.4	1.04	0	3.05	0.91	0	3.61	0.16	0	1.19	0.83	0	3.27	0.63	0	3.16
0.4–0.5	0.46	0	2.66	0.39	0	2.11	0.05	0	0.63	0.22	0	1.36	0.20	0	1.18
0.5–0.6	0.13	0	1.19	0.15	0	1.81	0.02	0	0.65	0.11	0	1.04	0.10	0	1.27
0.6–0.7	0.08	0	0.71	0.06	0	0.61	0.01	0	0.52	0.06	0	0.64	0.03	0	0.66
0.7–0.8	0.01	0	0.63	0.01	0	0.58	0	0	0	0.04	0	0.66	0.02	0	0.62
0.8–0.9	0.02	0	0.53	0.02	0	0.57	0	0	0	0.01	0	0.65	0	0	0.46
0.9–1	0.01	0	0.62	0	0	0.45	0.01	0	0.49	0.01	0	0.68	0	0	0.47

### Classification of power ramps for each month

**Figure 15:**
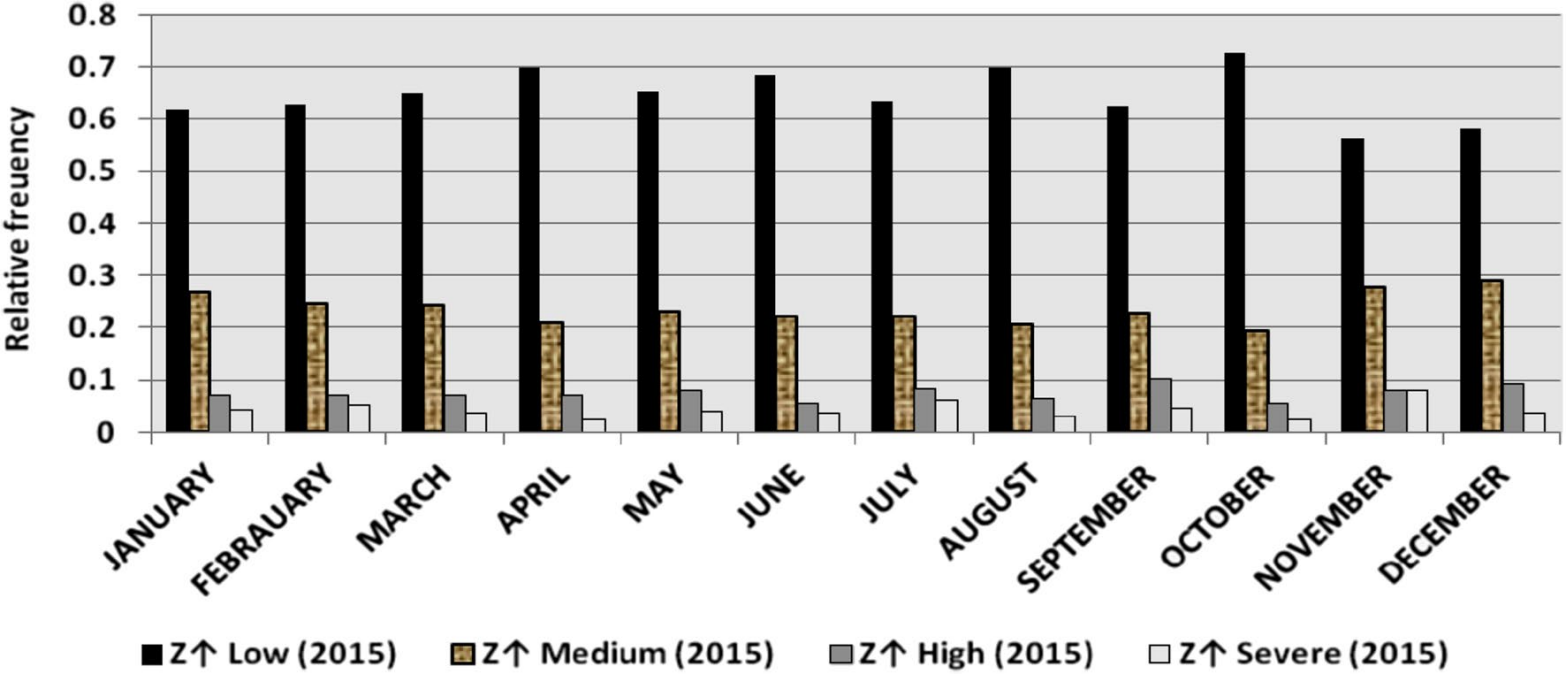
Classification of upward ramps for each month in 2015 according to the standard deviation scores.

**Figure 16:**
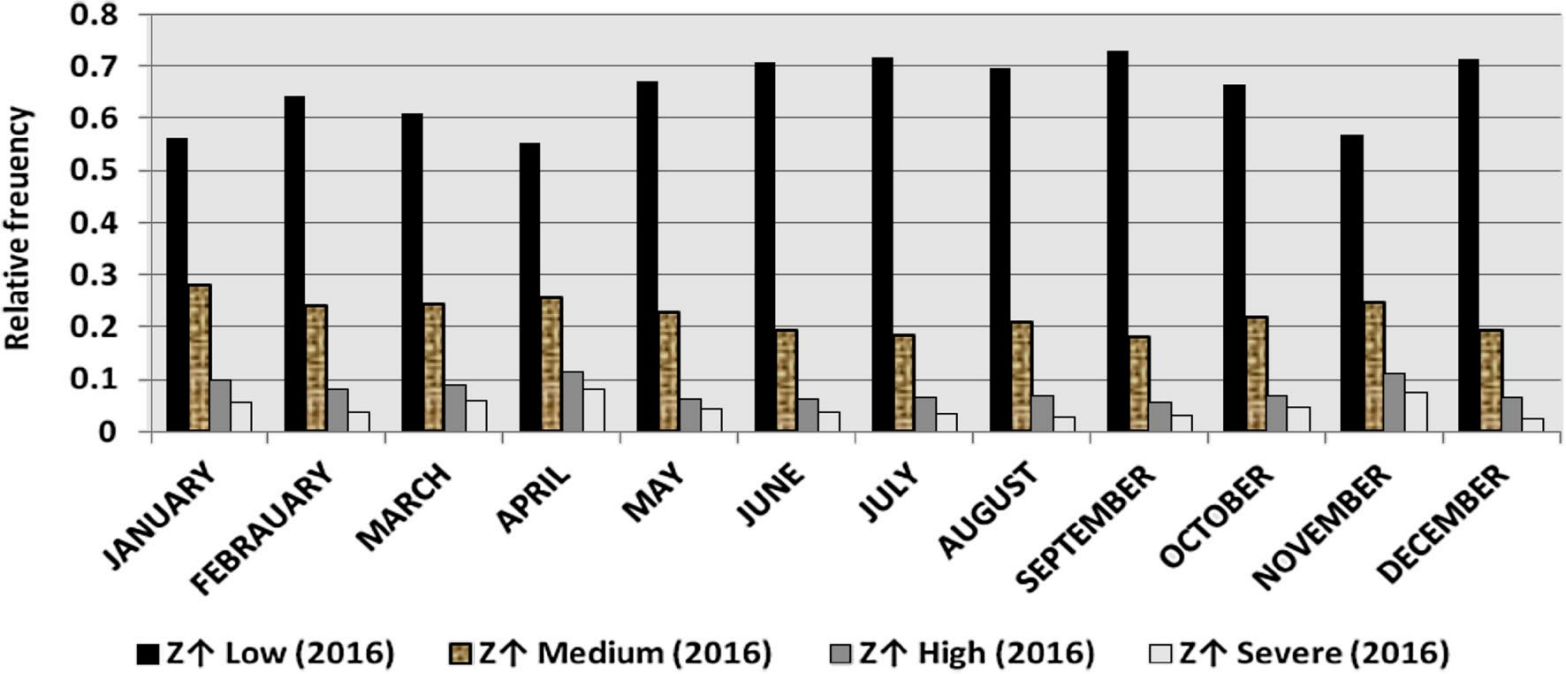
Classification of upward ramps for each month in 2016 according to the standard deviation scores.

**Figure 17:**
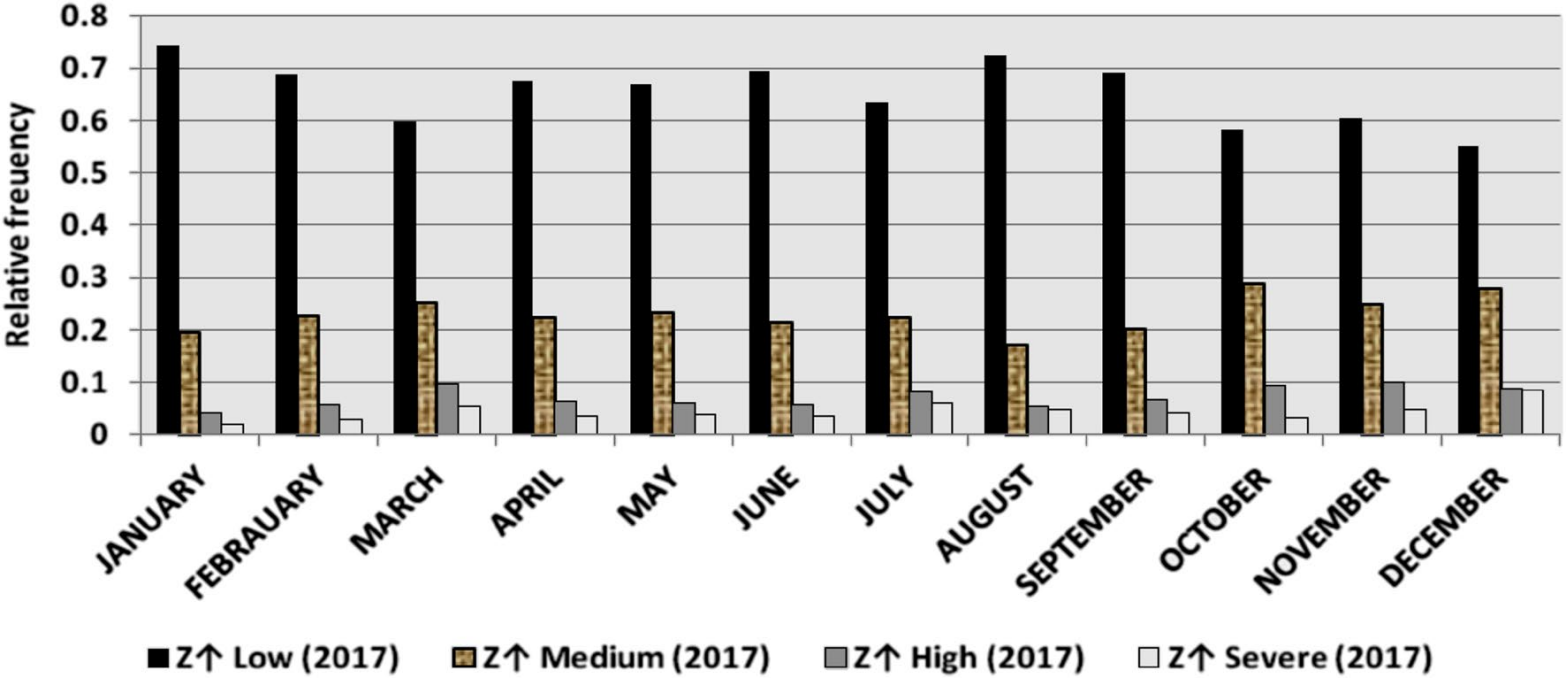
Classification of upward ramps for each month in 2017 according to the standard deviation scores.

**Figure 18:**
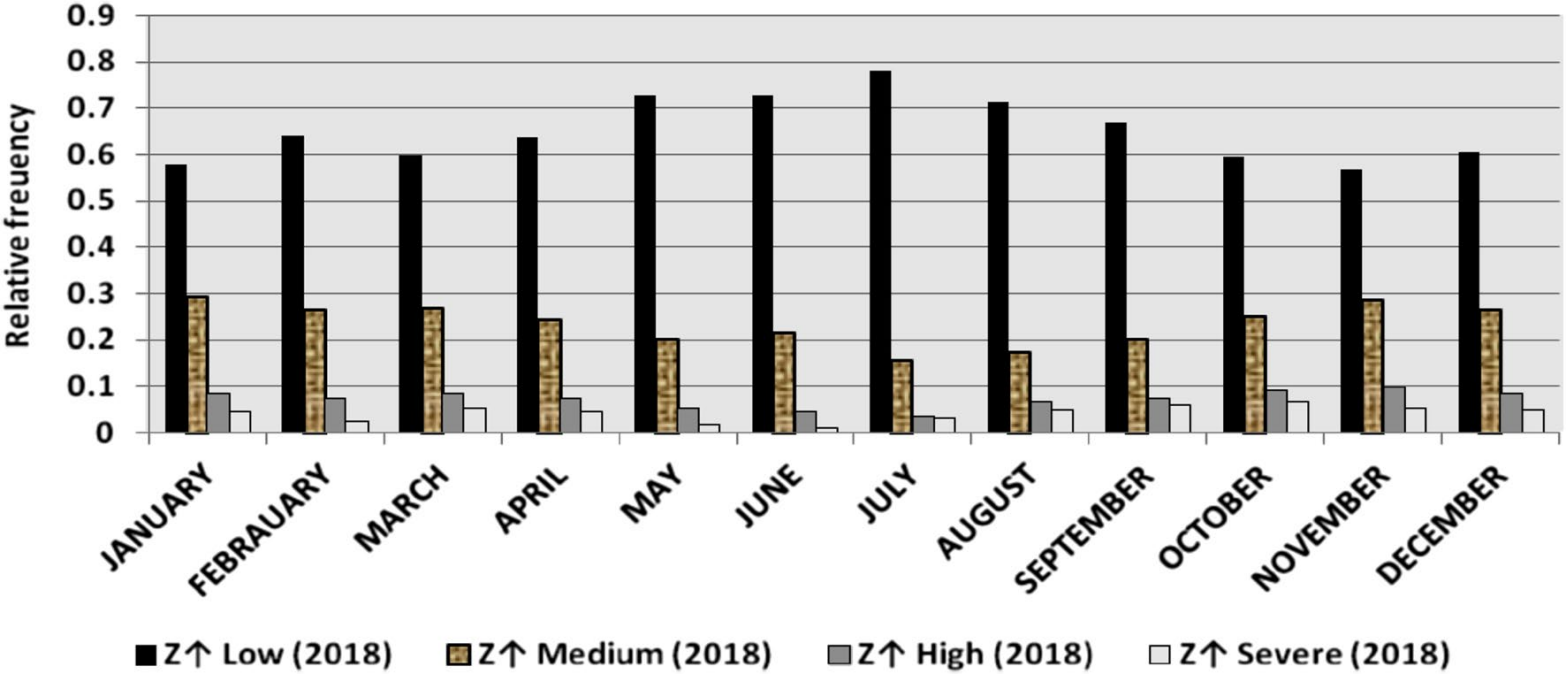
Classification of upward ramps for each month in 2018 according to the standard deviation scores.

**Figure 19:**
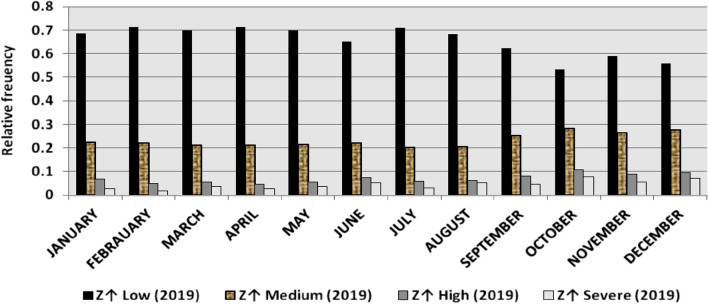
Classification of upward ramps for each month in 2019 according to the standard deviation scores.

**Figure 20:**
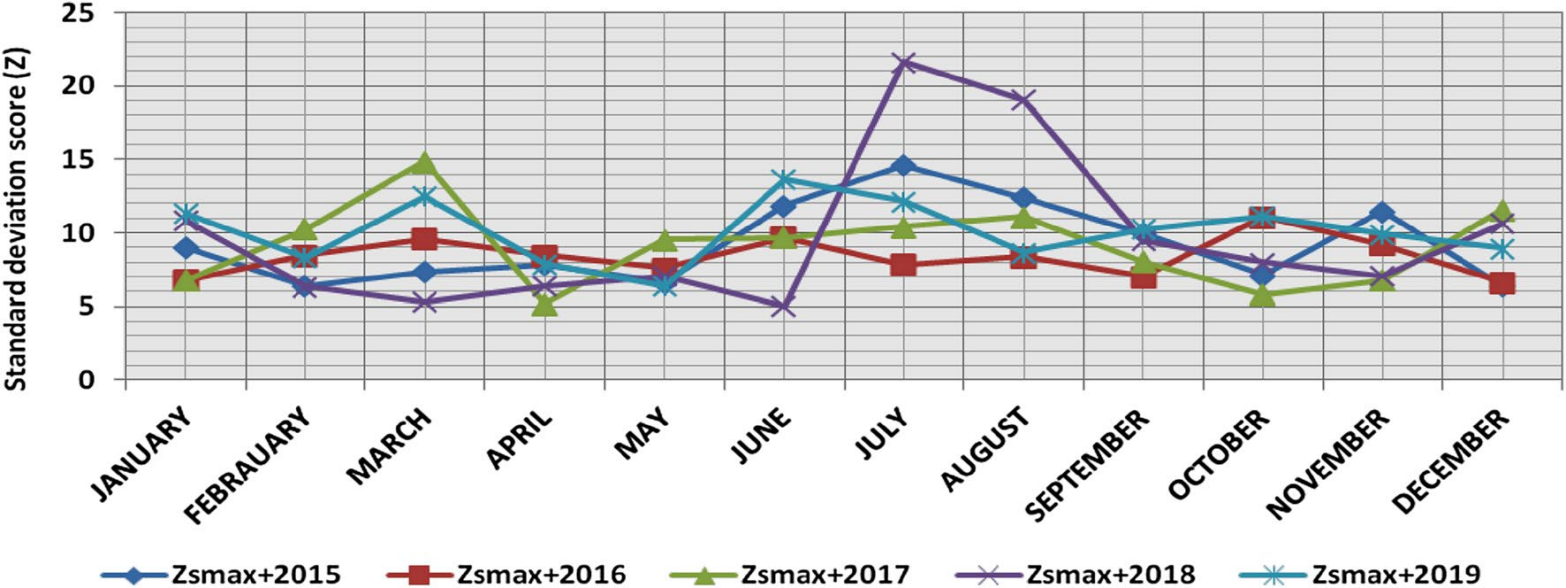
Comparison of standard deviation scores for the maximum upward ramps for each month in the 5 years.

The classification results demonstrate the differences between months in the levels of power ramps, as the first proposed technique illustrates the following:

(i) For upward ramps, comparing the results of the 5 years illustrates the following:
  October had the highest relative frequency of severe power ramps in 2018 and 2019, and in general, severe upward power ramps were common in October, November, and March.
  The relative frequencies of each category of power ramps overall months in the 5 years are as follows:
  Low ramps ranged from 53.5 to 77.8%, with an average percentage ranging from 64.6 to 65.5%, medium ramps ranged from 15.4 to 29.3%, with an average percentage ranging from 22.28 to 23.59%, high ramps ranged from 3.57 to 11.3%, with an average percentage ranging from 6.9 to 7.8%, severe ramps ranged from 1.08 to 8.34%, with an average percentage ranging from 4.19 to 4.61%, high plus severe ramps ranged from 4.64 to 19.9%, with an average percentage ranging from 11.3 to 12.4%.
  The average relative frequency of each category of power ramps overall months in the 5 years is within a very narrow band.
  The classification results of downward ramps are presented in Figs. 15, 16, 17, 18 and 19 and Table 8. Figure 20 compares the standard deviation scores of maximum upward power ramps within a time interval of 15 min for each month in the 5 years, which ranged from 5 to 21.7, with their average values ranging from 8.5 to 10 overall months in the 5 years
(ii) For downward ramps, comparing the results of the 5 years illustrates the following:
  Severe downward power ramps were common in November, December, and January.
  The relative frequencies of each category of power ramps overall months in the 5 years are as follows:
  Low ramps ranged from 53.6 to 78.1%, with an average percentage ranging from 64.45 to 65.86%, medium ramps ranged from 15.5 to 29.2%, with an average percentage ranging from 22.87 to 23.73%, high ramps ranged from 3.75 to 11.57%, with an average percentage ranging from 7.07 to 7.45%, severe ramps ranged from 1.72 to 8.76%, with an average percentage ranging from 4.1 to 4.48%, high plus severe ramps ranged from 5.47 to 19.79%, with an average percentage ranging from 11.27 to 11.89%.
  The average relative frequency of each category of power ramps overall months in the 5 years is within a very narrow band.

**Figure 21:**
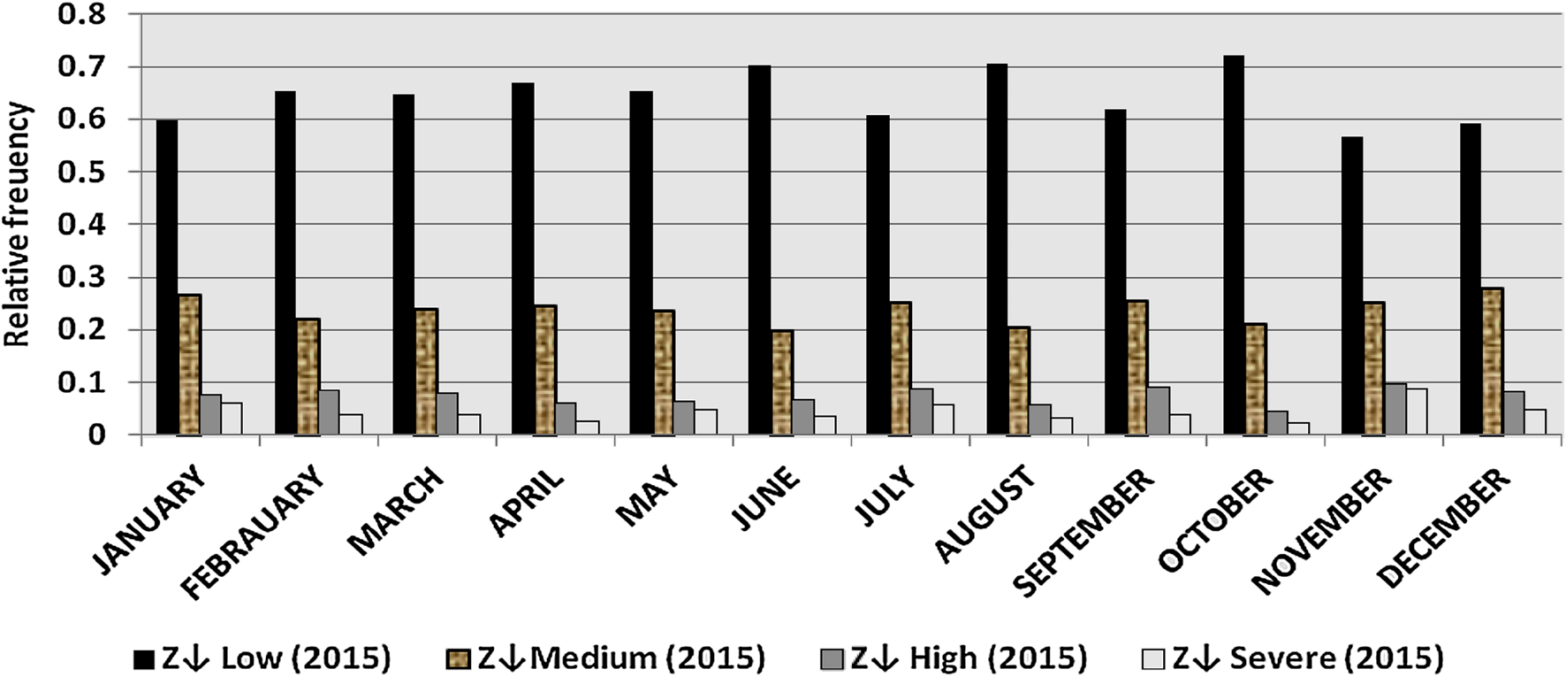
Classification of the downward ramps for each month in 2015 according to the standard deviation scores.

**Figure 22:**
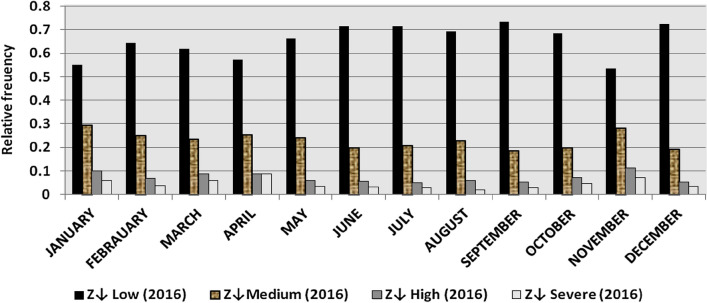
Classification of the downward ramps for each month in 2016 according to the standard deviation scores.

**Figure 23:**
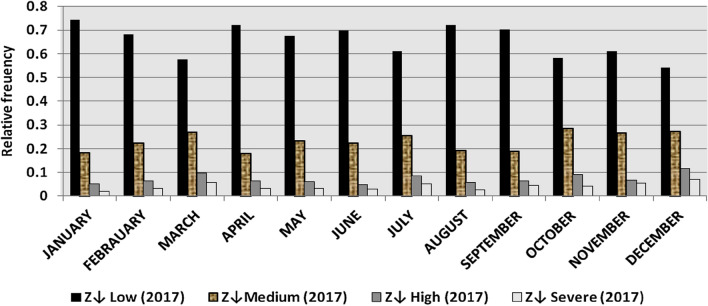
Classification of the downward ramps for each month in 2017 according to the standard deviation scores.

**Figure 24:**
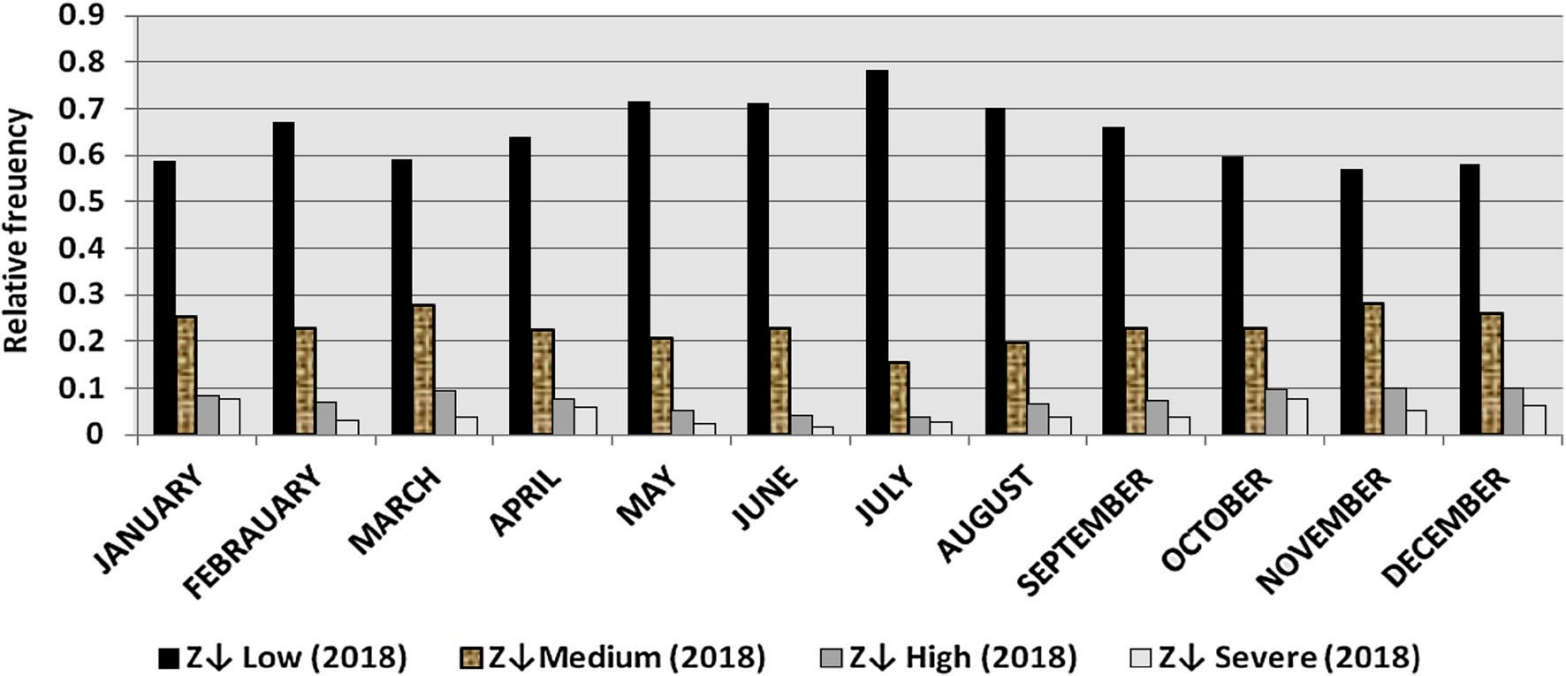
Classification of the downward ramps for each month in 2018 according to the standard deviation scores.

**Figure 25:**
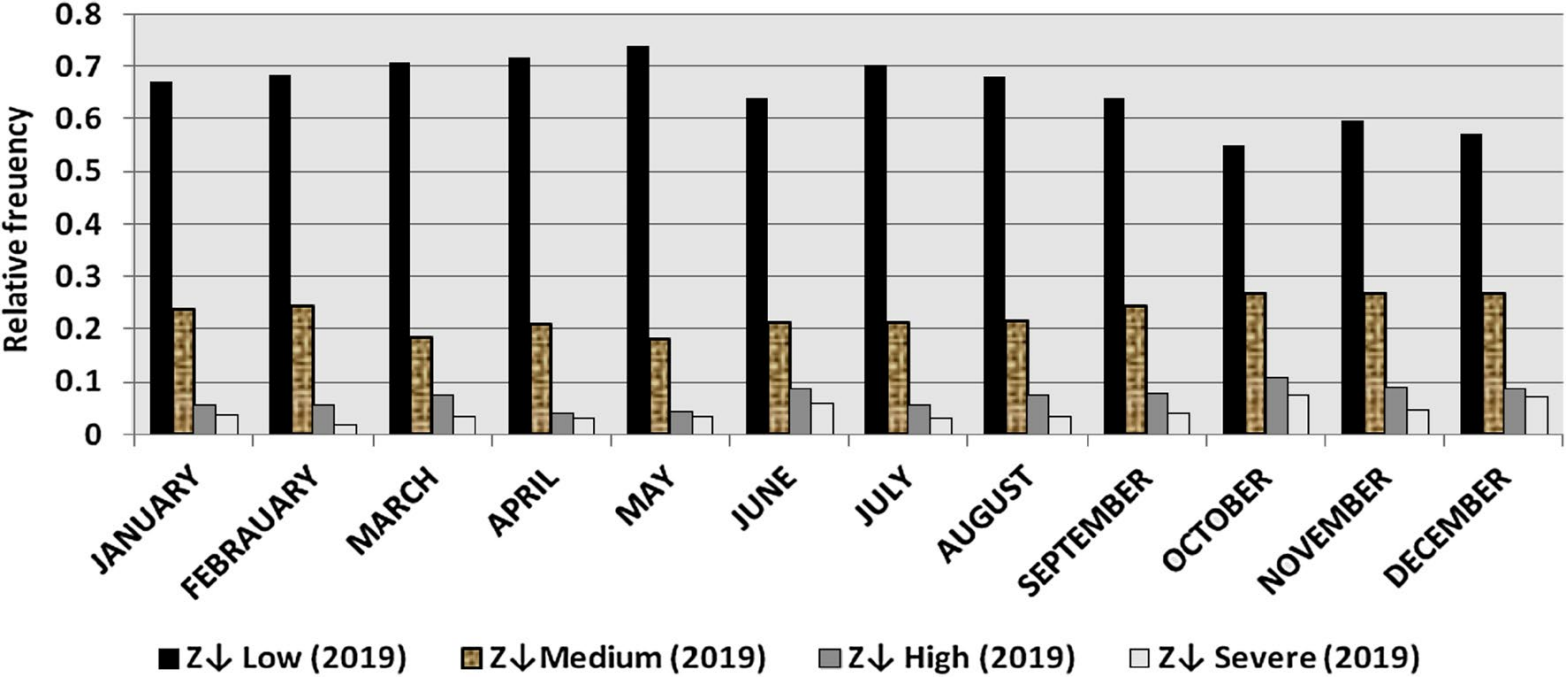
Classification of the downward ramps for each month in 2019 according to the standard deviation scores.

**Figure 26:**
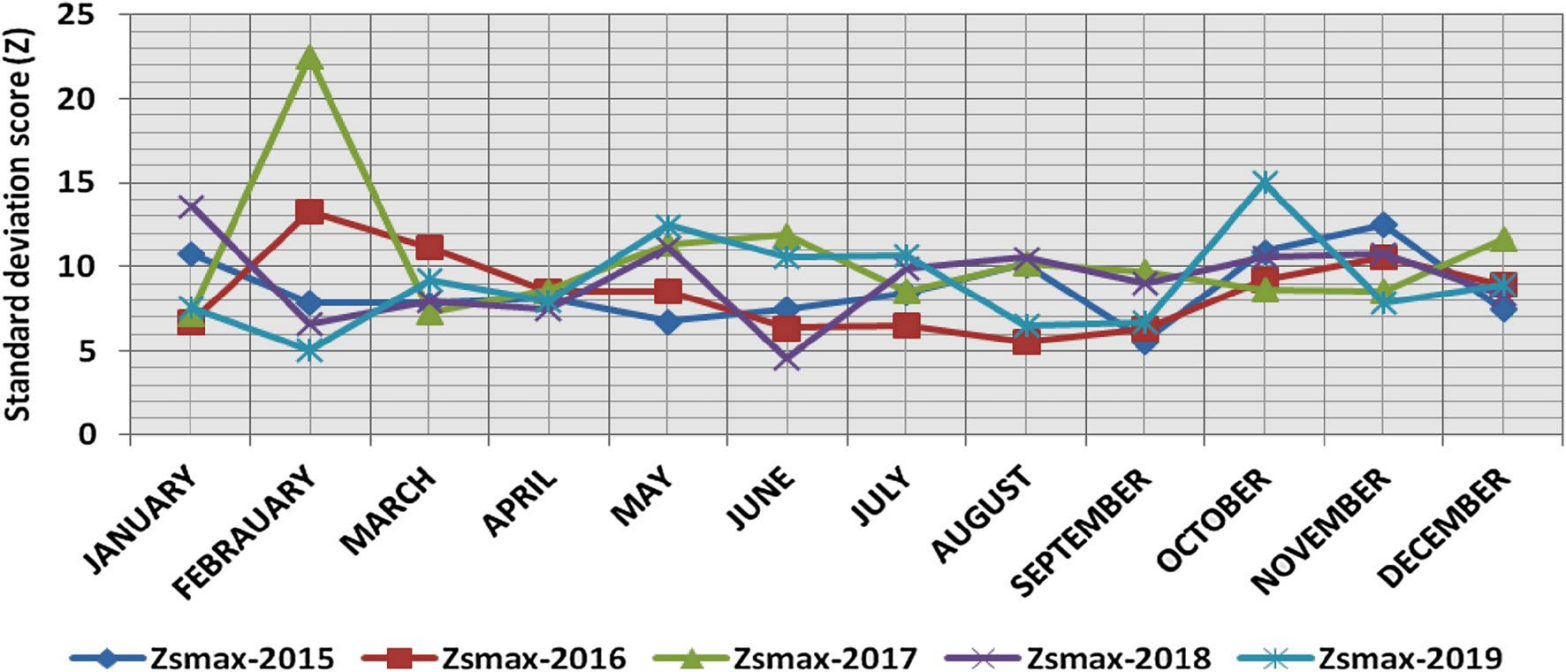
Comparison of the standard deviation scores for maximum downward ramps for each month in the 5 years.

The classification results are presented in Figs. 21, 22, 23, 24 and 25 and Table 8. Figure 26 compares the standard deviation scores of maximum downward power ramps within a time interval of 15 min for each month in the 5 years, which ranged from 4.5 to 22.5, with their average values ranging from 8.5 to 10.8 overall months in the 5 years.

**Table 8:** The range and the average relative frequency as a percentage of each category of power ramps overall months in the 5 years according to the standard deviation score classification.

Ramp category	2015			2016			2017			2018			2019		
	Avg.	Range		Avg.	Range		Avg.	Range		Avg.	Range		Avg.	Range	
		From	To		From	To		From	To		From	To		From	To
Upward power ramps															
Low	64.61	56.38	72.55	65.32	55.20	72.98	65.52	55.07	74.40	65.28	56.77	77.82	65.50	53.51	71.50
Medium	23.59	19.39	28.92	22.28	18.23	28.10	22.88	17.05	28.83	23.38	15.41	29.30	23.19	20.29	28.11
High	7.49	5.49	10.14	7.80	5.58	11.30	7.21	4.16	9.98	7.15	3.57	9.80	6.94	4.68	10.62
Severe	4.31	2.39	7.92	4.61	2.53	7.89	4.38	2.05	8.34	4.19	1.08	6.63	4.37	1.63	7.76
Downward power ramps															
Low	64.45	56.54	72.29	65.38	53.62	73.36	65.62	54.26	74.47	65.08	56.84	78.14	65.86	54.94	74.07
Medium	23.73	19.66	27.97	22.96	18.46	29.22	23.08	18.03	28.40	23.03	15.50	27.84	22.87	18.27	26.93
High	7.42	4.48	9.61	7.18	5.17	11.08	7.20	4.92	11.57	7.45	3.75	10.12	7.07	4.07	10.93
Severe	4.40	2.21	8.76	4.48	1.91	8.71	4.10	2.07	6.90	4.44	1.72	7.70	4.20	1.80	7.29

**Figure 27:**
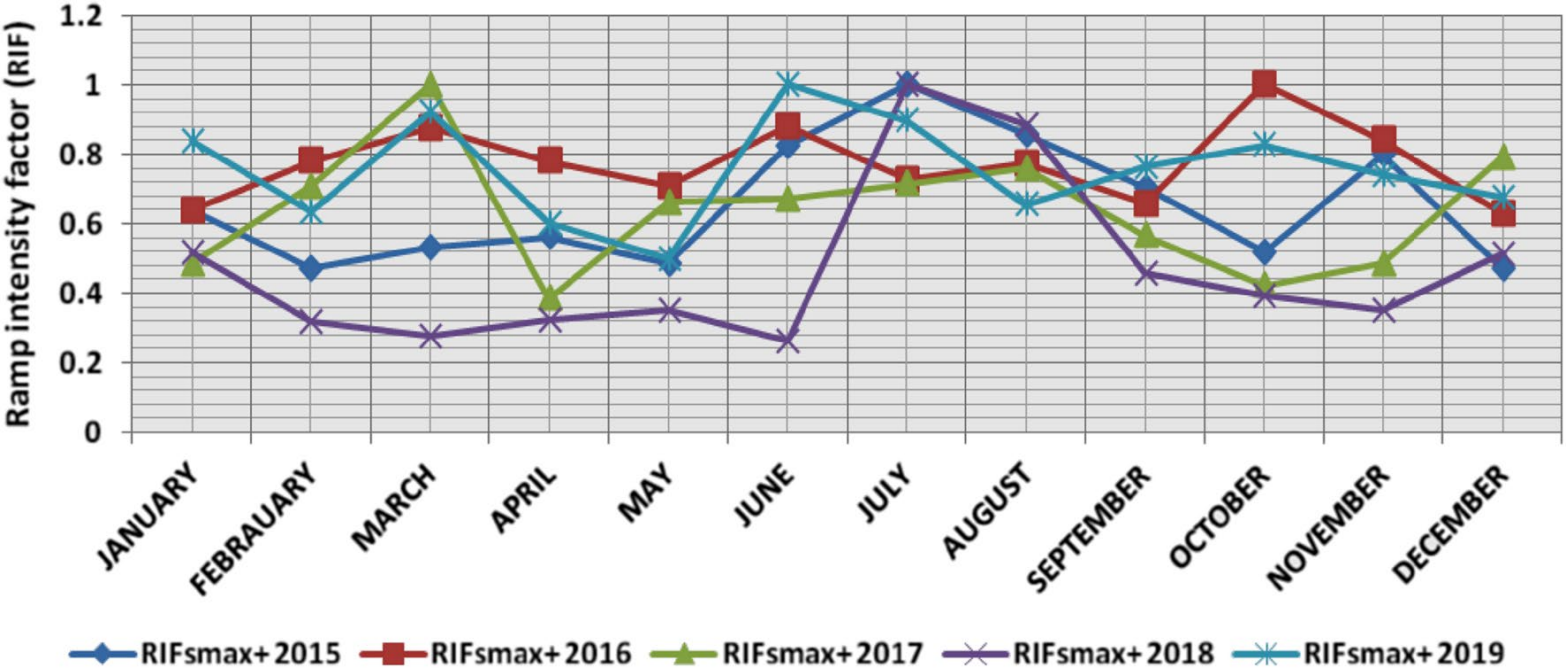
Comparison of RIFs for maximum upward power ramps for each month in the 5 years.

**Figure 28:**
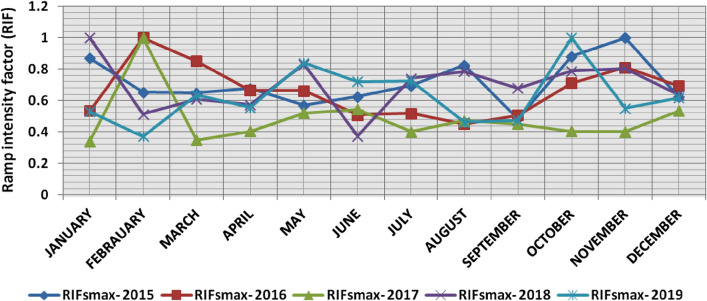
Comparison of RIFs for maximum downward power ramps for each month in the 5 years.

The results of the second proposed technique illustrate the following:

(i) *For upward ramps* The most severe upward power ramps with RIF from 0.8 to 1 occurred mostly in March, June, July, August, and October. In the 5 years, the RIF of 1 repeated twice in March and July, and the RIF of upward ramps in February, April, May, September, and December did not exceed 0.8. Figure 27 shows the comparison of ramp intensity factors of the maximum upward ramps for each month in the 5 years.
(ii) *For downward ramps* The most severe downward power ramps with RIF from 0.8 to 1 occurred mostly in January, February, May, October, and November. In the 5 years, the RIF of 1 repeated twice in February, and the RIF of downward ramps in April, and September did not exceed 0.7, while they did not exceed 0.8 in June and July. Figure 28 shows the comparison of ramp intensity factors of the maximum downward ramps for each month in the 5 years.

### Classification of power ramps for each season

**Figure 29:**
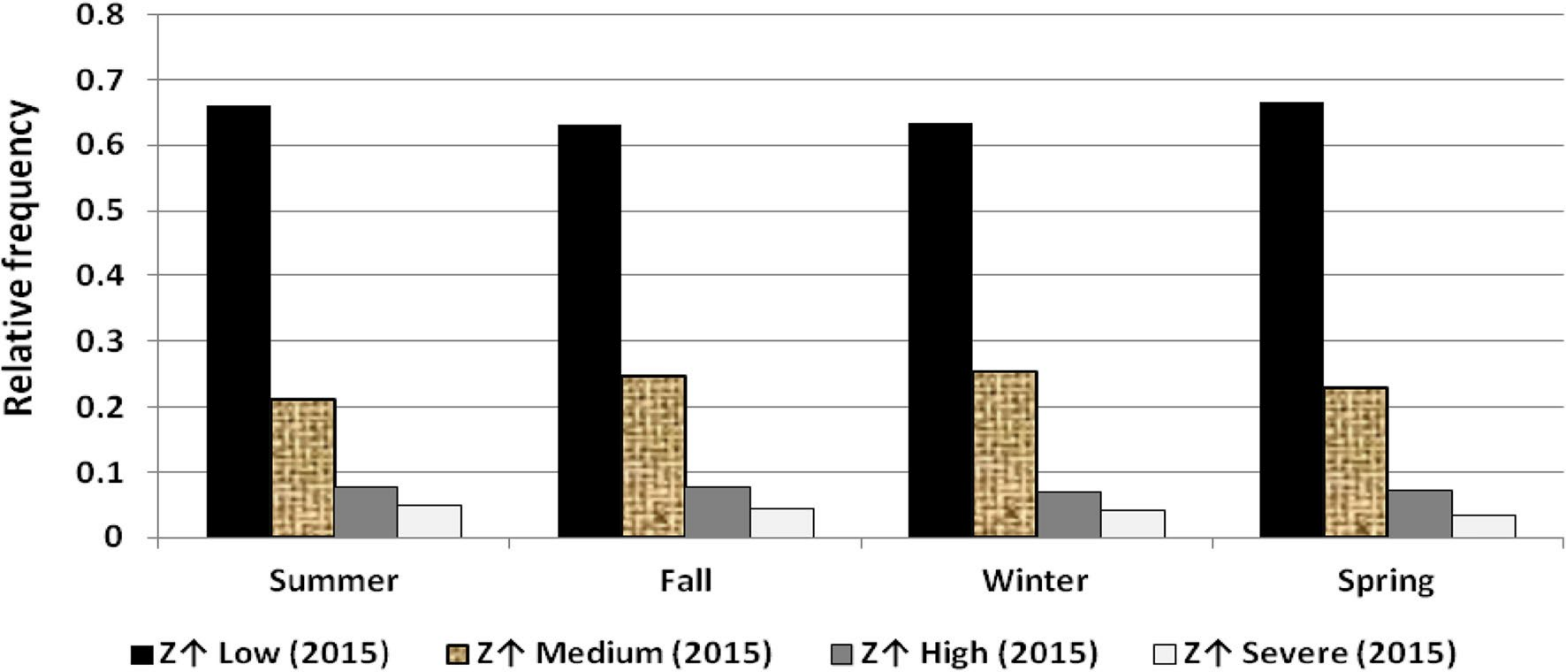
Seasonal classification of upward power ramps in 2015 according to the standard deviation scores.

**Figure 30:**
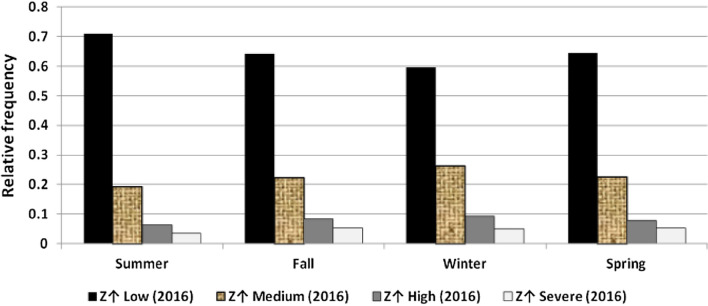
Seasonal classification of upward power ramps in 2016 according to the standard deviation scores.

**Figure 31:**
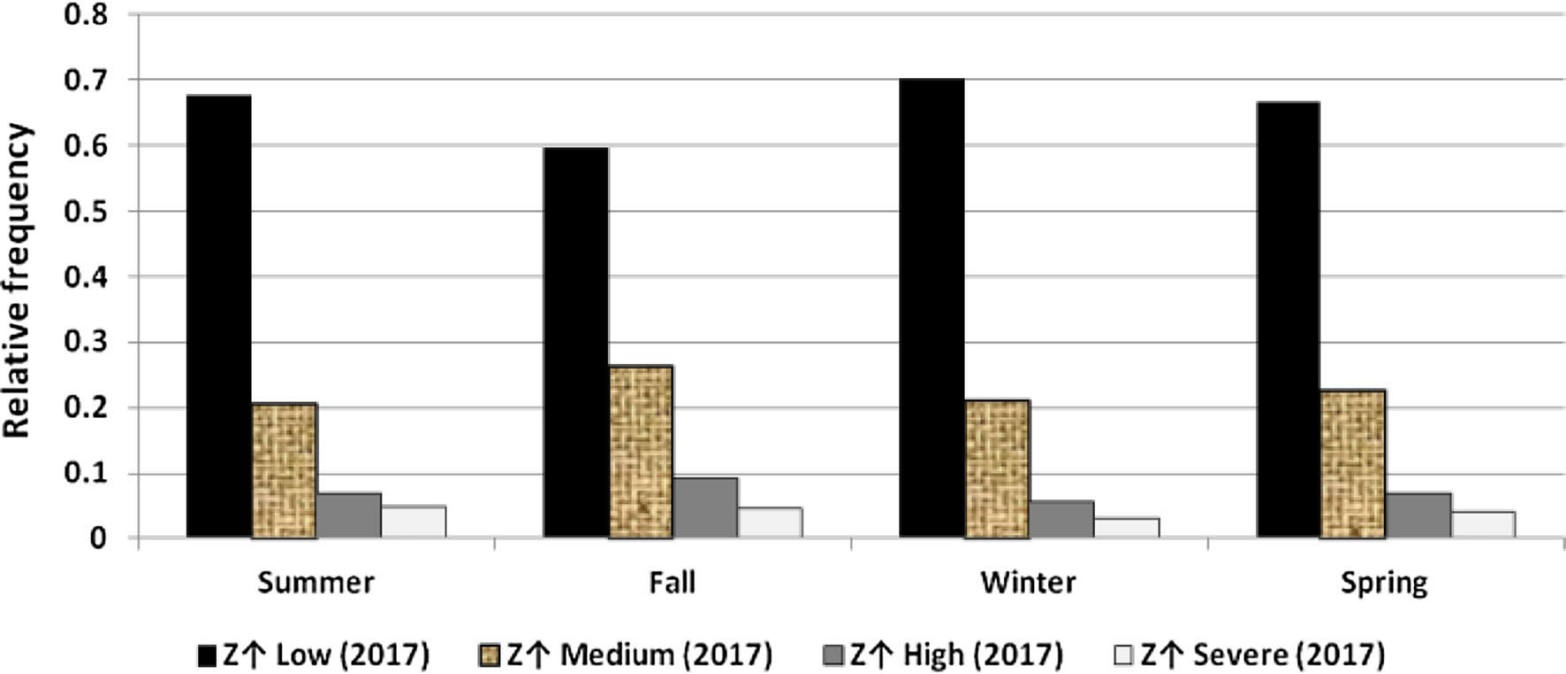
Seasonal classification of upward power ramps in 2017 according to the standard deviation scores.

**Figure 32:**
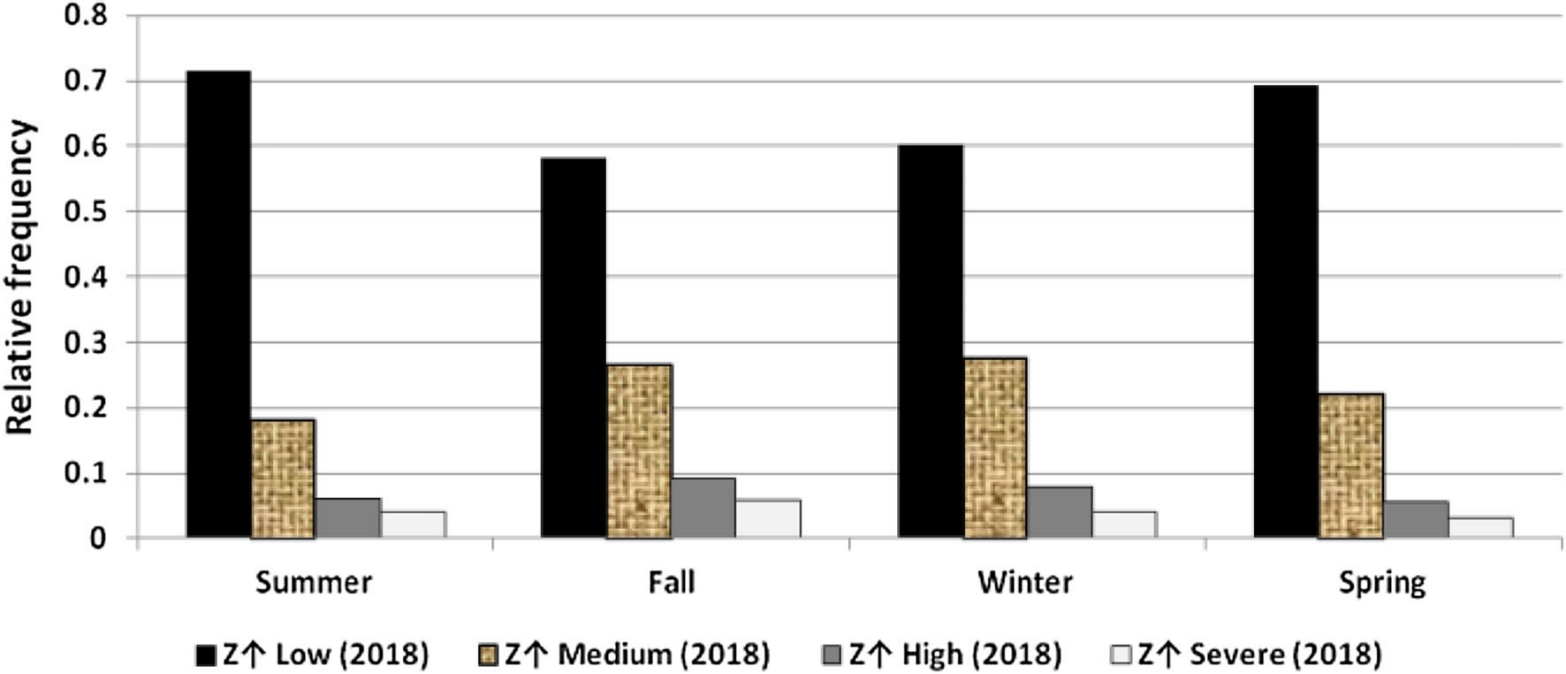
Seasonal classification of upward power ramps in 2018 according to the standard deviation scores.

**Figure 33:**
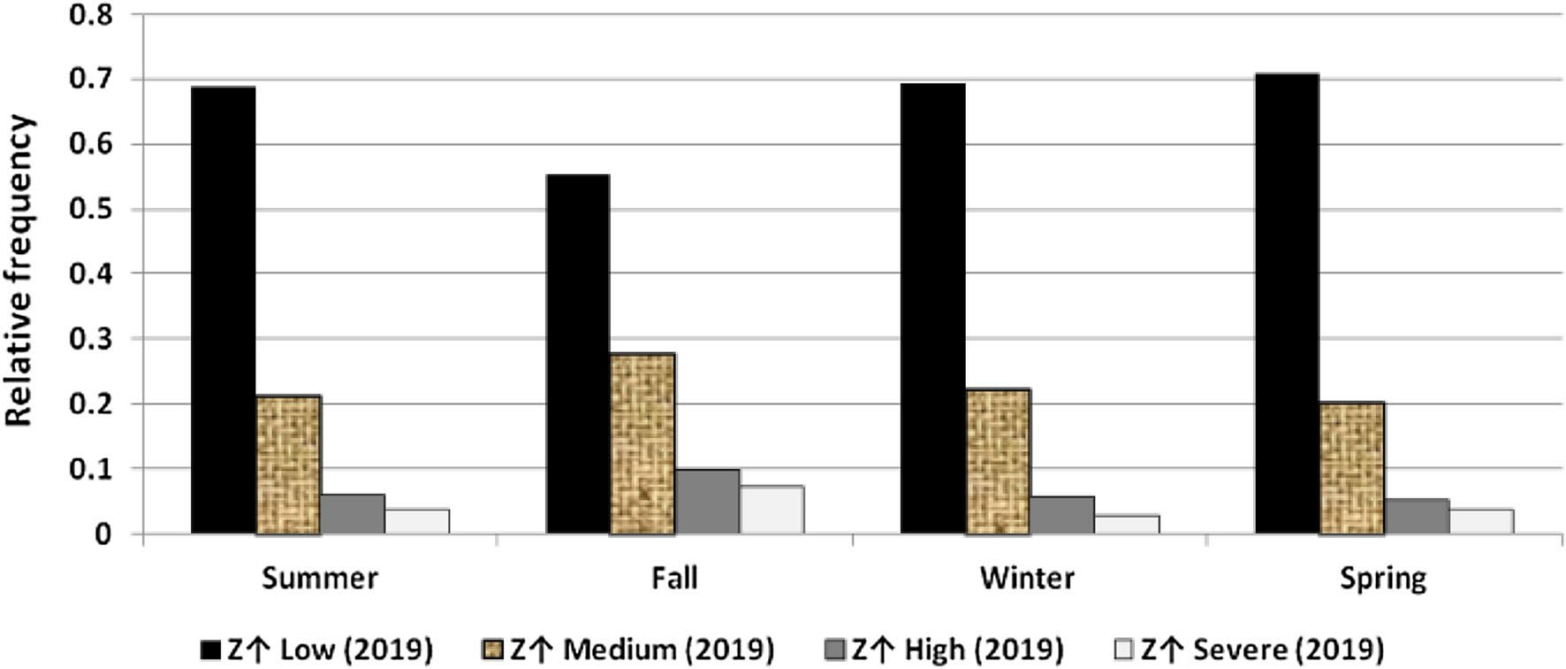
Seasonal classification of upward power ramps in 2019 according to the standard deviation scores.

**Figure 34:**
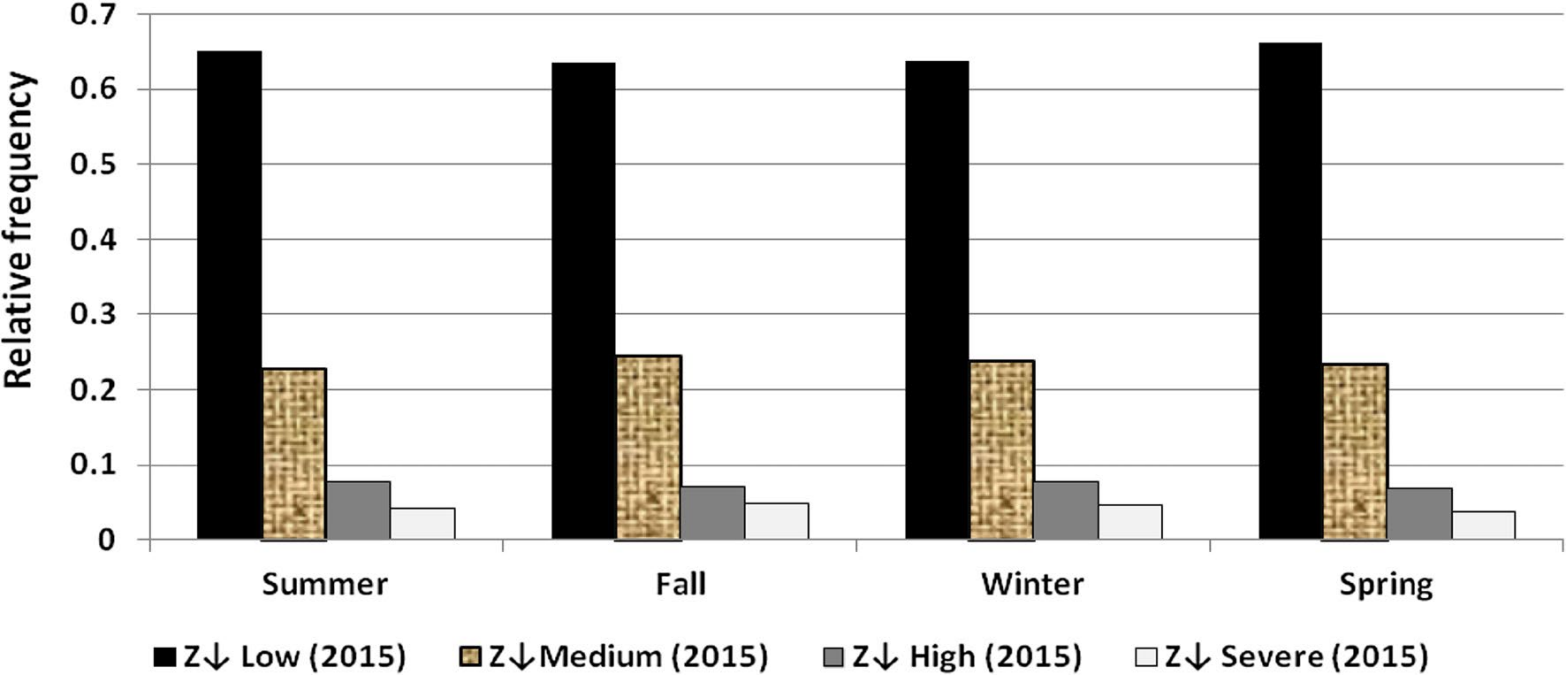
Seasonal classification of downward power ramps in 2015 according to the standard deviation scores.

**Figure 35:**
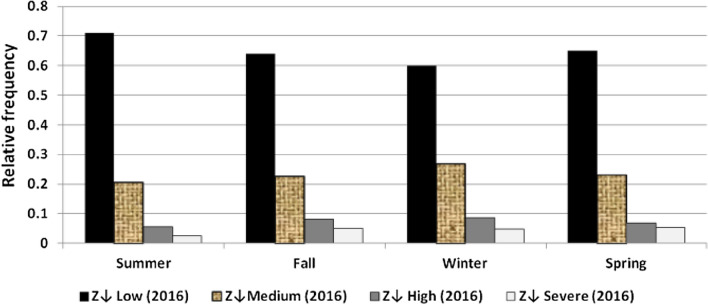
Seasonal classification of downward power ramps in 2016 according to the standard deviation scores.

**Figure 36:**
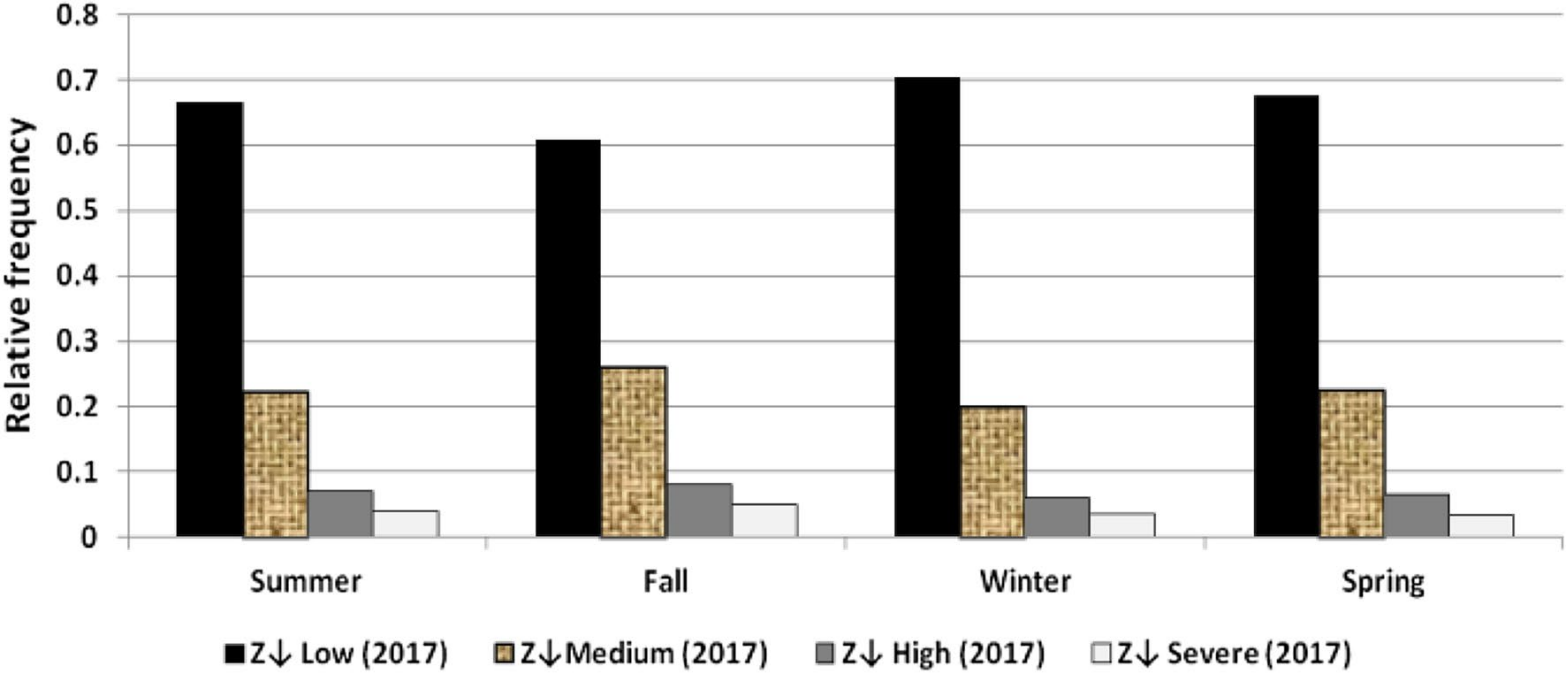
Seasonal classification of downward power ramps in 2017 according to the standard deviation scores.

**Figure 37:**
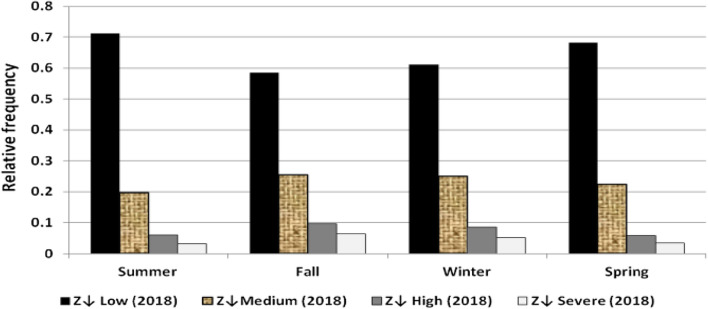
Seasonal classification of downward power ramps in 2018 according to the standard deviation scores.

**Figure 38:**
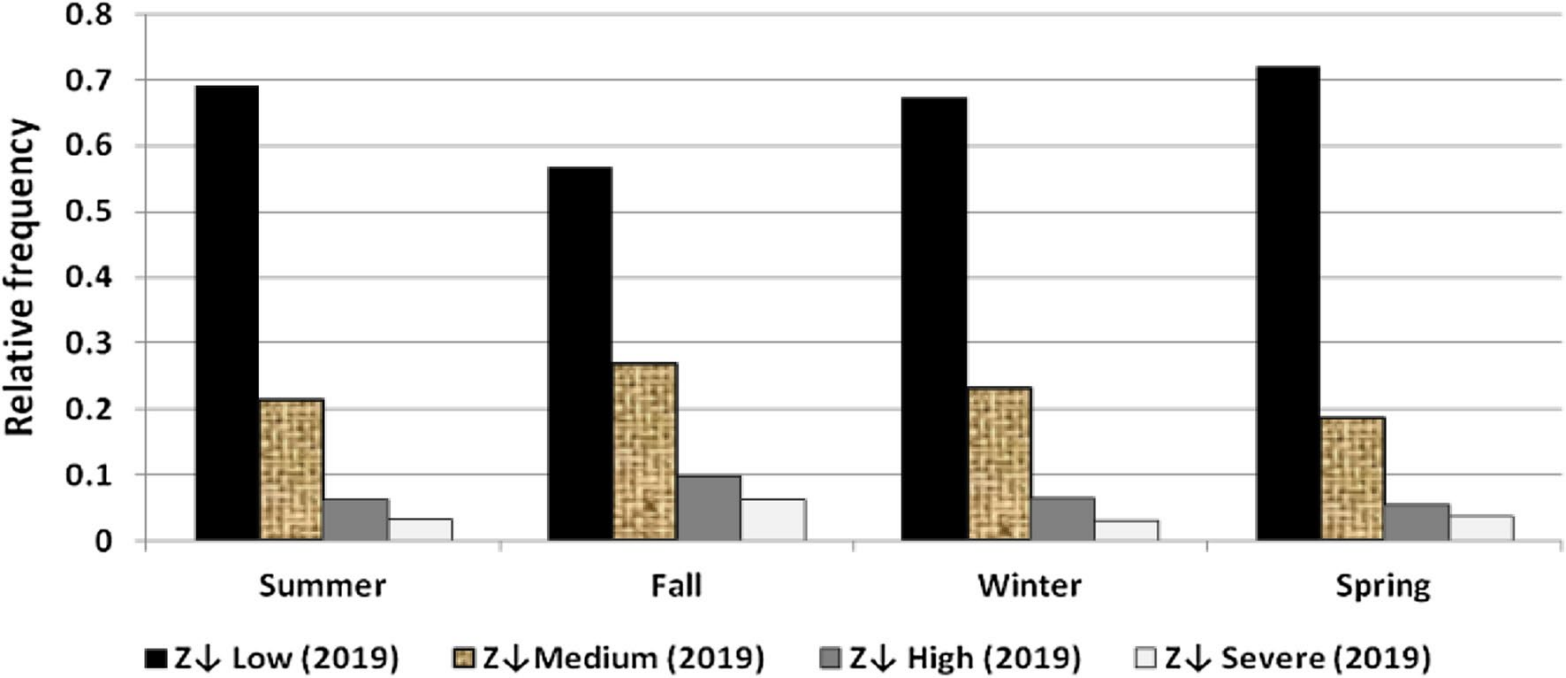
Seasonal classification of downward power ramps in 2019 according to the standard deviation scores.

The results illustrated the seasonal characteristics of wind power ramps as the results of the first proposed technique in Figs. 29, 30, 31, 32, 33, 34, 35, 36, 37 and 38 illustrated that severe power ramps of both types occurred mostly in the fall and severe upward power ramps were less common in winter and spring. In the summer, severe downward power ramps were less common. While the results of the second proposed technique illustrated the following:

- For upward ramps, the RIF of more than 0.5 were most common in summer and less common in winter.
- For downward ramps, the RIF of more than 0.5 were most common in the fall and less common in the summer.

### Yearly classification of power ramps

**Figure 39:**
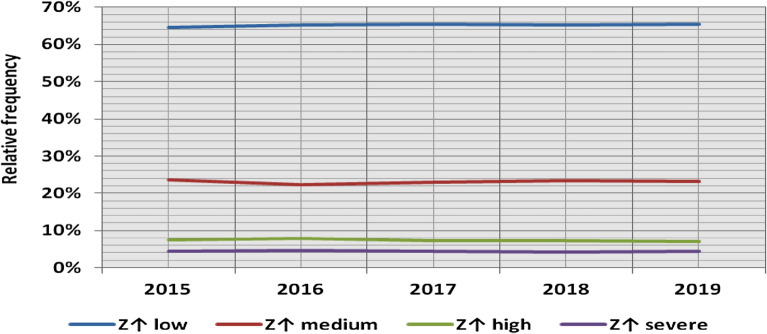
The yearly classification of upward power ramps according to the standard deviation scores.

**Figure 40:**
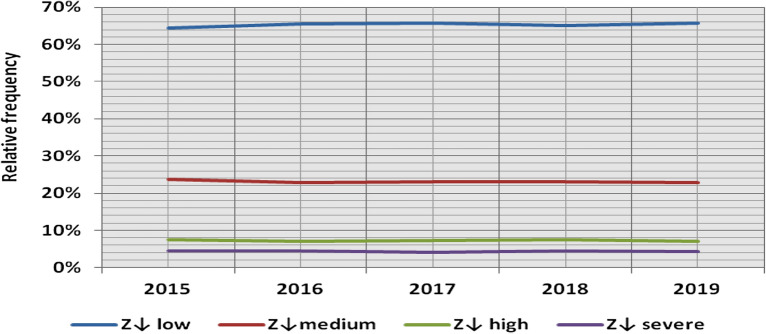
The yearly classification of downward power ramps according to the standard deviation scores.

The classification results illustrated the yearly features associated with wind power ramps, as the results of the first proposed classification technique for upward and downward power ramps show that the 5 years have the same percentage for each ramp category, as the relative frequency of low, medium, high, and severe power ramps in the 5 years represents 65%, 23%, 7%, and 4% of the total power ramps, respectively, as shown in Figs. 39 and 40. The class borderlines are dependent on both the average and standard deviation of power ramps and as previously demonstrated by ramp characteristic indicators (RCI)^47^, they have a nearly fixed percentage of the average wind installation, resulting in a nearly constant relative frequency of each class.

The classification results of the second proposed technique for upward and downward power ramps show that the RIF of the majority of power ramps in the 5 years is less than 0.3, as it is not less than 97.6% for upward power ramps and 98.3% for downward power ramps. Table 9 presents the relative frequency for each category of upward power ramps in the 5 years, which illustrates that the relative frequency of upward power ramps that have a high RIF of more than 0.5 over the 5 years ranged from 0.029 to 0.378%, with an average of 0.19%, and ramps that have a high RIF of more than 0.7 over the 5 years ranged from 0.012 to 0.092%, with an average of 0.052%. while Table 10 presents the relative frequency for each category of downward power ramps in the 5 years; which illustrates that the relative frequency of downward power ramps that have a high RIF of more than 0.5 over the 5 years ranged from 0.028 to 0.242%, with an average of 0.177%, and ramps that have a high RIF of more than 0.7 over the 5 years ranged from 0.006 to 0.051%, with an average of 0.033%.

**Table 9:** The relative frequency for each category of upward power ramps in the 5 years based on RIFs.

Year	0–0.1 (%)	0.1–0.2 (%)	0.2–0.3 (%)	0.3–0.4 (%)	0.4–0.5 (%)	0.5–0.6 (%)	0.6–0.7 (%)	0.7–0.8 (%)	0.8–0.9 (%)	0.9–1 (%)
2015	83.22	13.25	2.59	0.58	0.21	0.09	0.02	0.02	0.02	0.01
2016	74.91	17.72	4.97	1.47	0.55	0.23	0.06	0.05	0.03	0.01
2017	84.31	12.17	2.50	0.69	0.19	0.08	0.04	0.03	0.00	0.01
2018	92.62	6.28	0.83	0.19	0.05	0.01	0.01	0.00	0.01	0.01
2019	83.37	12.54	2.89	0.70	0.27	0.10	0.05	0.04	0.02	0.02
Average	83.69	12.39	2.75	0.73	0.25	0.10	0.04	0.03	0.01	0.01
Minimum	74.91	6.28	0.83	0.19	0.05	0.01	0.01	0.00	0.00	0.01
Maximum	92.62	17.72	4.97	1.47	0.55	0.23	0.06	0.05	0.03	0.02

**Table 10:** The relative frequency for each category of downward power ramps in the 5 years according to RIFs.

Year	0–0.1 (%)	0.1–0.2 (%)	0.2–0.3 (%)	0.3–0.4 (%)	0.4–0.5 (%)	0.5–0.6 (%)	0.6–0.7 (%)	0.7–0.8 (%)	0.8–0.9 (%)	0.9–1 (%)
2015	78.51	16.20	3.57	1.02	0.45	0.12	0.07	0.01	0.02	0.01
2016	81.55	13.88	3.06	0.91	0.37	0.14	0.06	0.01	0.02	0.01
2017	93.15	5.88	0.73	0.16	0.05	0.02	0.01	0.00	0.00	0.01
2018	81.42	14.25	3.05	0.84	0.21	0.11	0.06	0.03	0.01	0.01
2019	84.95	11.82	2.23	0.63	0.20	0.10	0.03	0.02	0.01	0.01
Average	83.92	12.41	2.53	0.71	0.26	0.10	0.05	0.01	0.01	0.01
Minimum	78.51	5.88	0.73	0.16	0.05	0.02	0.01	0.00	0.00	0.01
Maximum	93.15	16.20	3.57	1.02	0.45	0.14	0.07	0.03	0.02	0.01

## Conclusion

The flexibility of the power system is linked to the ability to deal with the power ramps in the net load curve in real-time at various operational stages. However, as the installations of renewable energy continue growing, the ability to manage these fluctuations has become a difficult task. While wind and PV generations are largely dependent on wind speed and solar irradiance respectively, both speed and irradiance variations cannot fully represent the variability of power and the percentage of forecast error is considerable. Therefore, getting the range and the level of power ramps in the system, especially in wind and solar generation, is becoming vital information in the operation of power systems. The majority of previous research was primarily based on a binary classification of ramp events which assumes that ramp events are equally identical, which in reality is not true, where different control actions are applied according to the level of such variations. Additionally, as there is no agreement on a precise definition of the ramp event, different threshold values have been considered and the number of ramp events identified in these researches was highly sensitive to the selected threshold value, which increased the difficulty of comparing results from them. The other studies were based on an arbitrary classification of ramp events, in which a randomized and non-causative classification was used to classify ramp events into several levels. As a result of this heterogeneity in the classification of ramp events, a unified framework for solving this complexity is required. Two new techniques for classifying power ramps have been introduced in this paper to overcome the drawbacks of previous techniques. The classification techniques are based on the statistical analysis of historical power ramps that occurred within a selected time interval. The time interval is chosen by the power system operator according to the operating stage under consideration. In the first technique, the power ramps have been classified based on their average value and standard deviation, while the power ramps in the second have been classified based on their maximum value. The new classification techniques have been applied to the output power of Belgium’s aggregated wind farms from 2015 to 2019 for the following time horizons: (i) Historical data of power ramps at each observation time to provide detailed information on the level of the power ramps at each observation time t, (ii) Historical data of daily power ramps to provide detailed information on the level of power ramps in certain months, seasons, or years. This information includes the relative frequency of a certain class or degree of power ramp, which can be used to estimate the probability of occurrence of different categories of power ramps, as well as the times when high and severe ramps are most likely to occur, in order to take the required precautions to balance these ramps in the event of a significant forecast error, thus enhancing the system’s flexibility. The results obtained by comparing the 5 years demonstrated that the class borderlines using the first technique are dependent on both the average and standard deviation of power ramps, and as previously demonstrated by RCI in Ref.^49^, they have a nearly fixed percentage of the average wind installation, resulting in a nearly constant relative frequency of each ramp category, despite the fact that the average installed wind capacity has been doubled; as the relative frequency of low, medium, high, and severe power ramps in the 5 years represents 65%, 23%, 7%, and 4% of the total power ramps, respectively. This classification, on the other hand, is dynamic, as it is a function of the average and standard deviation. As a result, a power ramp classified as severe one year may be classified as medium the next. The fixed percentages of RCI can be utilized to classify power ramps in real-time. The high standard deviation value demonstrated that the power ramps’ magnitude extended over a wider range. So, in the second technique using the RIF, each ramp within the studied time interval Δt has been assigned a degree, indicating the intensity of that ramp in relation to the maximum value of historical power ramps within the same time interval. Accordingly, the two proposed classification methods are complementary. The power ramps classification and analysis improve understanding of variability in renewable generation. The two new classification methods have the following advantages over the previous methods: they overcome the drawbacks of previous techniques, they discriminate between different ramp events, and they introduce a unified and causative framework for ramp event classifications, allowing comparison of results from different systems. They directly provide valuable information that, in addition to forecasted information, can be used to determine the appropriate amounts of spinning and standing reserves to reduce the cost and difficulty of absorbing variability. The future work will include the application of the new classification approaches to other variable renewable generations.

## Data Availability

The datasets analysed during the current study of aggregated wind farms’ output power recorded every 15 min in Belgium are available in (Wind-power generation (elia.be))^48^.
